# Supplementary material for: Psychometric properties of the Malay version of the Behavioural Regulation in Exercise Questionnaire (BREQ-3)
Source: PLoS One. 2022 Jun 24;17(6):e0269099. doi: 10.1371/journal.pone.0269099 (PMC9231722; doi:10.1371/journal.pone.0269099)
Supplement: S1 BREQ data — (PDF) [file pone.0269099.s001.pdf]

ID	GENDER	AGE	RACE	DISEASES_	SMOKING_	DURATION_	C1	C2
1	1	23	1	2	2	240	4	0
2	1	20	4	2	2	180	4	1
3	1	20	1	2	2	300	3	3
4	2	19	7	1	2	120	4	0
5	1	20	1	2	2	300	3	0
6	2	20	2	2	2	140	2	2
7	2	19	1	2	2	120	4	0
8	2	21	1	2	2	90	4	0
9	2	19	1	2	2	60	3	2
10	2	19	1	2	2	60	2	0
11	2	21	2	2	2	180	3	1
12	2	19	1	2	2	20	2	0
13	2	23	11	2	2	180	4	1
14	2	19	2	2	2	270	3	0
15	2	20	8	2	2	240	4	0
16	2	19	1	2	2	30	2	1
17	2	20	1	2	2	120	3	0
18	2	20	7	2	2	360	3	1
19	2	19	1	2	2	120	4	1
20	2	20	2	2	2	0	3	2
21	2	21	9	2	2	180	3	0
22	2	20	2	2	2	180	0	1
23	1	20	2	2	2	120	3	2
24	2	20	2	2	2	120	3	1
25	2	20	2	2	2	120	3	0
26	2	20	2	2	2	140	2	2
27	2	19	1	2	2	45	3	0
28	2	19	2	2	2	140	2	1
29	2	21	2	2	2	100	2	1
30	2	20	2	2	2	360	4	0
31	2	19	2	2	2	210	3	0
32	2	19	5	2	2	80	4	0
33	2	20	1	2	2	90	2	1
34	2	20	1	2	2	150	2	1
35	2	20	1	2	2	120	4	0
36	2	20	5	2	2	360	4	0
37	2	20	1	999	999	45	2	3
38	1	20	1	2	2	135	3	1
39	1	20	1	2	2	270	4	0
40	1	19	1	2	2	25	3	2
41	1	20	6	2	2	240	4	0
42	2	19	5	2	2	120	4	0
43	2	20	2	2	2	120	3	2
44	2	19	2	2	2	140	2	2
45	2	20	1	2	2	60	3	0
46	2	19	1	2	2	60	3	0
47	2	19	1	2	2	40	3	1
48	2	19	1	2	2	90	4	1
49	2	20	1	2	2	120	2	1

50	2	19	1	2	2	30	2	1
51	2	20	1	2	2	60	2	3
52	2	20	1	2	2	180	2	3
53	2	19	1	2	2	720	3	0
54	2	20	1	2	2	540	4	1
55	2	21	1	2	2	90	4	0
56	2	19	1	2	2	60	3	0
57	2	21	1	2	2	120	4	0
58	2	20	1	2	2	120	4	0
59	2	20	1	2	2	60	4	1
60	2	19	1	2	2	120	2	0
61	2	20	1	2	2	90	3	1
62	2	20	1	2	2	45	3	1
63	2	19	1	2	2	180	3	2
64	2	19	1	2	2	300	2	1
65	2	21	1	2	2	45	4	0
66	2	21	1	2	999	0	2	2
67	2	19	1	2	2	30	4	0
68	2	21	1	2	2	120	3	0
69	2	19	1	2	2	30	3	1
70	2	20	1	2	2	180	4	1
71	2	19	1	2	2	30	3	1
72	2	20	1	2	2	120	4	1
73	2	19	1	2	2	70	4	1
74	2	19	1	2	2	60	3	1
75	2	20	1	2	2	50	4	0
76	2	19	1	2	2	135	4	0
77	2	20	1	2	2	450	4	0
78	2	20	1	2	2	180	3	3
79	2	20	1	2	2	360	4	0
80	2	21	1	2	2	60	4	0
81	2	20	1	2	2	120	2	0
82	2	19	1	2	2	60	4	1
83	2	19	1	2	2	140	4	1
84	2	20	1	2	2	90	4	1
85	2	20	1	2	2	90	4	0
86	2	20	1	2	2	60	4	0
87	2	20	1	2	2	90	2	1
88	2	20	1	2	2	80	3	0
89	1	19	1	1	2	90	2	2
90	2	19	1	2	2	210	4	4
91	2	19	1	2	2	60	4	3
92	2	20	10	2	2	180	3	1
93	2	19	1	2	2	60	4	0
94	2	19	1	1	2	80	3	0
95	2	19	1	2	2	120	1	1
96	2	21	1	2	2	360	4	1
97	2	20	11	2	2	60	3	0
98	1	20	1	2	2	300	4	0
99	2	22	2	2	2	60	3	1

100	2	21	2	2	2	40	3	2
101	2	20	2	2	2	480	2	1
102	2	19	3	2	2	150	4	1
103	2	22	1	2	2	150	4	0
104	2	22	1	2	2	360	2	1
105	2	22	1	2	2	60	4	0
106	2	22	1	2	2	0	3	0
107	2	23	1	2	2	450	3	0
108	2	20	1	2	2	240	4	0
112	2	20	1	2	2	60	1	1
113	2	20	1	2	2	0	3	1
116	2	20	1	2	2	240	4	0
120	1	21	1	2	2	30	4	1
122	2	21	1	2	2	60	2	3
123	2	21	999	2	2	45	3	2
124	2	20	1	2	999	450	3	1
128	2	20	1	2	2	245	4	0
129	2	20	1	2	2	60	3	2
148	2	23	2	999	999	60	3	0
149	2	23	2	2	2	60	3	0
150	2	21	2	2	2	30	2	2
151	1	21	2	2	2	60	2	1
152	1	21	2	2	2	480	3	1
153	1	21	2	2	2	180	2	3
154	2	20	2	2	2	120	3	1
155	1	20	2	2	2	180	3	1
156	2	20	2	2	2	270	3	1
158	2	20	3	2	2	30	4	0
161	1	20	3	999	999	0	3	3
174	2	21	1	2	2	90	4	0
195	2	21	1	2	2	30	3	2
196	2	21	1	2	2	45	2	3
197	2	21	1	2	2	45	2	2
199	2	21	1	2	2	60	3	2
206	2	21	1	2	2	30	1	1
208	2	23	1	2	2	60	4	1
209	2	24	1	2	2	90	4	0
210	2	21	1	1	2	60	4	3
211	2	21	1	2	2	180	4	0
212	2	21	1	2	2	0	3	3
213	2	22	1	2	2	120	3	0
214	2	23	1	2	2	120	3	1
215	2	24	1	2	2	0	3	1
216	2	21	1	2	2	0	3	1
217	2	23	1	2	2	0	3	1
218	2	21	1	2	2	0	3	1
219	2	23	1	2	2	0	4	2
220	2	21	9	2	2	120	4	0
225	2	20	1	999	2	180	4	1
226	2	20	1	2	2	60	3	0

227	2	20	1	2	2	60	2	1
228	2	20	1	2	2	60	4	1
229	2	20	1	2	2	50	3	1
233	2	20	1	2	2	30	2	0
234	2	20	1	2	2	100	3	0
237	2	20	1	2	2	120	4	0
238	1	20	1	2	2	540	3	1
239	1	20	1	2	2	90	3	0
240	1	20	1	2	2	120	3	1
241	1	20	1	2	2	90	3	3
242	1	20	1	2	2	600	2	0
243	1	20	1	2	1	720	1	2
244	1	20	1	2	2	900	4	0
246	1	20	1	2	2	120	3	2
247	1	20	1	2	2	70	4	0
248	1	20	1	1	1	60	3	0
249	1	20	1	2	2	540	3	0
250	1	20	1	999	999	30	4	1
255	2	20	1	2	2	30	4	1
258	2	20	1	2	2	120	3	0
267	2	20	1	2	2	60	3	0
268	2	20	11	2	2	140	3	0
269	2	21	1	2	2	60	4	0
270	2	22	1	2	2	150	2	0
271	2	21	1	999	999	15	3	1
273	2	20	1	2	2	30	4	0
274	2	20	1	2	2	540	3	0
275	2	20	1	2	2	180	4	1
276	2	20	1	2	2	120	3	1
277	2	20	1	2	2	90	4	1
278	2	20	1	2	2	60	2	1
279	2	20	1	2	2	60	3	3
302	2	19	1	2	2	90	3	2
303	2	19	1	2	2	630	4	1
304	2	19	1	2	2	135	2	1
305	2	19	1	1	1	90	3	1
309	2	20	1	2	2	90	4	0
310	2	19	1	2	2	45	3	0
311	1	20	1	2	2	60	4	0
313	2	20	1	2	2	120	3	4
314	2	20	1	2	999	120	3	4
315	2	21	2	2	2	0	3	2
316	2	21	2	2	2	240	3	2
317	2	20	1	2	2	840	3	0
318	2	21	1	2	2	30	3	1
319	2	21	2	2	2	120	3	2
320	2	22	1	2	2	0	3	3
321	2	22	1	2	2	0	4	0
322	2	22	1	2	2	0	2	1
323	2	22	1	2	2	0	2	0

324	2	22	1	2	2	60	4	4
325	2	22	1	2	999	0	4	2
326	2	22	1	1	2	180	4	1
327	2	22	1	2	2	0	4	0
338	2	20	1	2	2	120	4	0
339	2	22	1	2	2	120	4	0
343	2	22	1	2	2	120	2	0
344	2	22	1	2	2	840	4	0
345	2	22	1	1	2	20	2	0
347	2	22	1	2	2	60	2	1
348	2	22	1	1	1	60	2	0
349	2	22	1	1	2	360	4	0
352	2	24	1	2	2	180	4	3
360	2	20	3	2	2	30	3	2
370	2	20	3	2	2	600	3	2
371	2	20	3	2	2	120	3	2
373	1	23	2	2	2	180	2	3
376	1	21	2	2	2	60	2	0
385	2	20	1	2	2	30	2	1
386	2	19	1	999	999	100	4	0
387	2	19	1	2	2	40	3	2
388	2	20	2	2	2	90	4	1
389	2	20	2	2	2	240	3	1
399	2	20	7	2	2	225	3	0
403	2	23	1	2	2	450	4	0
404	2	20	2	2	2	240	4	0
405	2	20	2	2	2	180	3	0
406	2	21	2	999	2	20	3	1
407	2	21	2	2	2	0	2	2
408	2	23	2	2	2	60	3	0
409	2	23	2	2	2	135	4	0
410	2	23	2	2	2	240	2	0
411	1	20	2	2	2	480	4	0
412	1	20	2	2	2	30	2	1
413	1	20	2	2	2	240	2	0
414	1	20	2	2	2	60	2	1
415	1	20	2	2	2	60	3	0
416	1	20	2	2	2	180	4	0
417	1	21	2	1	2	180	3	0
418	1	19	1	2	2	80	2	3
419	1	19	1	2	2	60	3	1
420	1	24	1	2	2	60	2	1
421	1	20	1	2	2	180	3	2
422	1	25	1	2	2	120	3	1
423	1	21	1	2	2	250	2	0
424	1	20	1	2	2	90	3	3
425	1	20	1	2	2	300	4	0
426	1	21	1	2	2	30	3	0
427	1	19	1	2	2	120	2	1
428	1	21	1	2	2	120	3	2

429	1	21	1	2	2	90	4	4
430	1	21	1	2	2	60	2	3
432	1	22	1	2	2	240	4	0
433	1	20	1	1	2	120	999	1
435	1	20	3	2	2	135	3	0
436	1	20	1	1	2	123	0	4
437	2	21	1	2	2	60	4	1
438	2	22	1	2	2	30	3	1
439	2	21	1	999	999	60	2	2
440	2	21	1	1	2	30	3	1
441	2	21	1	2	2	90	4	4
443	2	21	1	2	2	30	3	2
444	2	21	1	2	999	90	4	3
445	2	21	1	2	2	120	4	1
446	2	21	1	2	2	60	4	1
447	2	20	1	2	2	120	2	2
448	1	19	1	2	2	120	3	3
449	1	20	1	2	2	90	3	0
451	2	20	1	2	2	120	3	1
452	2	20	1	2	2	135	2	1
453	2	20	1	2	2	120	3	1
455	2	20	1	2	2	120	4	1
456	2	20	1	2	2	180	3	1
457	2	21	1	2	2	90	3	1
459	2	22	1	2	2	180	3	4
461	2	21	1	2	2	120	2	3
463	1	21	1	999	999	30	4	0
464	1	19	1	2	2	30	3	0
468	2	27	3	2	2	0	2	1
469	2	22	1	2	2	60	2	2
470	2	22	2	999	999	0	2	0
471	2	24	1	2	2	135	3	0
472	2	23	1	2	2	30	3	0
474	2	22	2	2	2	135	3	3
475	2	23	3	2	2	90	2	3
477	1	20	1	2	2	120	3	3
478	1	20	1	2	2	300	3	0
480	1	20	1	1	2	720	3	0
481	1	20	1	1	2	60	2	0
482	1	20	1	2	2	180	3	0
483	1	20	1	2	2	30	4	4
484	2	20	1	2	2	25	4	1
485	2	20	1	2	2	60	4	1
486	2	20	1	1	2	0	3	1
487	2	20	1	1	2	180	3	0
488	2	20	1	2	2	150	4	0
489	2	20	1	2	2	60	2	0
490	2	20	1	2	2	90	4	0
491	2	20	1	2	2	360	3	1
493	2	20	1	2	2	30	4	1

494	2	20	1	2	2	10	2	0
495	2	20	1	2	2	30	3	2
496	2	20	1	2	2	45	3	1
497	2	20	1	2	2	0	3	1
499	2	20	1	2	2	20	4	1
503	2	20	1	2	2	60	4	0
504	2	20	1	2	2	40	3	1
508	2	20	11	2	2	120	4	0
509	2	20	3	2	2	60	3	1
510	2	20	1	2	2	180	3	1
511	2	20	1	2	2	45	4	0
512	2	20	1	2	2	90	4	0
513	2	20	1	2	2	100	4	0
514	2	20	1	2	2	180	4	0
515	2	21	1	2	2	75	4	4
517	2	21	1	2	2	60	3	3
518	2	21	1	2	2	75	4	0
519	2	21	2	2	2	90	4	0
521	2	22	1	2	2	75	4	0
522	2	22	1	2	2	150	4	1
524	2	21	1	2	2	0	3	0
525	2	23	1	2	2	120	4	0
527	2	21	1	2	2	60	3	3
528	2	21	1	2	2	0	4	1
529	2	21	1	1	2	360	3	0
530	2	23	2	2	2	60	2	2
531	2	19	1	2	2	40	4	1
536	2	22	1	999	2	100	3	1
537	2	21	2	2	2	30	3	0
538	2	21	2	2	2	90	4	0
539	2	21	2	2	2	60	2	0
540	2	20	1	2	2	40	2	1
541	2	23	1	2	2	80	4	2
542	2	21	1	2	2	180	3	0
543	2	21	1	2	2	90	4	1
544	2	21	3	2	2	180	4	1
545	2	22	1	2	2	50	4	1
547	2	21	1	2	2	40	2	1
548	2	21	1	2	2	30	4	1
549	2	20	1	999	2	30	4	1
550	2	20	1	999	2	0	4	0
583	2	19	1	2	2	120	4	1
584	2	20	1	2	2	0	4	2
585	2	19	1	2	2	0	2	1
586	2	20	1	2	2	0	3	3
588	2	19	11	1	2	15	4	2
589	2	20	1	2	2	60	4	0
590	2	19	1	2	2	150	4	1
592	2	20	1	2	2	300	3	3
593	2	19	1	2	2	240	4	0

594	2	20	1	2	2	420	4	3
595	2	19	1	2	2	180	4	3
596	2	19	1	2	2	630	4	0
597	2	19	1	2	2	60	3	0
598	2	22	1	2	2	60	3	1
599	2	22	1	2	2	105	4	1
601	2	20	1	2	2	60	2	0
602	2	21	1	2	2	23	2	1
603	2	20	1	2	2	20	3	1
604	2	20	1	2	2	20	2	1
605	2	21	2	2	2	30	3	1
606	2	20	1	2	2	30	3	2
607	2	20	1	2	2	60	3	2
608	2	20	1	2	2	60	3	2
609	2	20	9	2	2	180	4	0
610	2	21	1	2	2	60	3	1
611	2	20	1	2	2	60	3	1
613	2	20	1	1	2	420	1	1
614	2	20	1	2	1	0	3	0
615	2	20	2	2	2	30	3	1
616	2	23	1	2	2	360	2	0
617	2	20	1	999	999	45	4	0
621	2	20	1	2	2	120	3	0
622	2	20	1	2	2	0	4	0
625	2	20	1	2	2	120	2	0
626	2	20	1	2	2	0	4	0
627	2	20	1	2	2	0	4	1
628	2	19	1	2	2	40	3	1
629	1	19	1	2	2	120	4	3
630	2	19	1	2	2	30	1	2
631	2	20	1	2	2	40	1	2
633	2	19	1	2	999	105	1	2
634	1	19	1	2	2	120	3	1
635	1	19	1	1	2	0	4	3
637	1	20	1	2	2	270	4	1
638	2	20	1	2	2	120	3	3
639	1	21	1	2	2	120	3	0
640	1	22	1	2	2	120	3	3
641	1	20	1	2	2	240	4	0
642	2	20	1	2	2	60	4	3
646	2	19	1	2	2	88	3	1
647	2	19	1	2	2	30	4	1
649	2	19	1	2	2	60	3	0
651	1	19	1	2	2	150	4	0
652	2	19	1	2	2	0	2	1
653	2	22	1	2	2	120	4	0
655	1	19	3	2	2	180	4	0
656	1	19	1	2	2	60	3	3
657	1	19	1	2	2	45	4	0
663	2	19	1	999	2	30	3	1

666	2	20	1	2	2	30	3	3
667	2	20	1	2	2	30	3	1
670	2	19	1	2	2	40	4	0
671	2	21	1	2	2	90	4	3
674	2	19	1	2	2	10	3	3
676	2	20	2	2	2	135	3	2
677	1	19	1	2	999	180	2	2
678	2	20	1	2	2	60	1	2
679	2	20	4	2	2	180	0	1
680	2	21	1	2	2	30	2	2
681	1	20	13	2	2	180	2	1
682	2	19	2	2	2	0	2	1
683	2	23	2	2	2	60	1	1
684	2	19	2	2	2	240	1	2
686	2	19	2	2	2	60	2	2
688	1	19	2	2	2	500	2	2
690	1	19	1	2	2	150	2	0
691	2	23	1	2	2	0	3	0
692	2	24	1	2	2	0	4	0
694	2	23	1	1	2	30	4	0
695	2	20	1	2	2	120	2	3
697	2	20	1	2	2	45	3	1
699	1	23	2	2	2	180	4	0
700	2	25	1	2	2	15	3	2
703	2	22	1	2	2	120	4	0
704	2	23	1	2	2	60	4	0
706	2	20	1	2	2	240	4	0
707	2	20	1	2	2	60	3	1
708	2	20	1	2	999	100	4	0
709	2	20	1	2	2	120	3	1
710	2	20	1	2	2	120	4	1
711	2	20	1	2	2	120	3	2
712	2	20	1	2	999	120	4	2
713	2	20	1	2	2	90	4	0
714	2	20	1	2	2	30	4	1
715	2	20	1	2	2	240	3	1
716	2	20	1	2	2	60	4	1
717	2	20	1	2	2	90	4	0
721	2	21	2	2	2	60	3	1
722	1	20	2	2	2	720	3	2
724	2	20	2	2	2	150	4	2
725	2	22	2	2	2	60	2	1
726	2	20	9	999	999	120	4	2
727	2	20	1	2	2	50	4	1
728	2	20	2	2	2	30	3	0
729	1	20	2	2	2	240	2	2
730	2	22	2	2	2	90	4	0
731	1	20	2	2	2	600	2	0
732	1	22	2	2	2	150	1	2
733	2	24	1	2	2	60	1	2

734	1	23	2	2	2	480	3	0
735	2	23	1	2	2	120	1	2
736	2	21	1	1	2	240	4	0
739	2	21	1	1	2	30	3	3
740	2	23	1	999	999	20	3	0
741	2	24	1	2	2	240	3	4
742	2	21	1	2	2	90	1	4
747	2	24	1	2	2	30	4	2
748	2	24	1	1	2	240	4	2
749	2	19	1	2	2	0	4	2
750	2	19	1	1	2	0	3	2
751	2	19	1	2	2	0	3	1
752	2	20	1	2	2	0	2	1
754	2	23	2	2	2	120	1	1
755	2	21	1	2	2	180	4	0
756	2	20	1	2	2	60	4	0
757	2	20	1	2	2	40	4	0
758	2	20	1	2	2	0	4	1
759	2	20	1	2	2	120	3	2
760	2	20	1	2	2	30	3	0
761	2	20	1	2	2	60	4	0
764	2	20	1	2	2	90	3	0
765	2	20	1	2	2	90	3	1
768	2	20	2	2	999	0	2	0
771	2	20	2	2	2	10	2	3
773	2	20	2	2	2	60	2	0
774	2	20	1	2	2	120	3	1
775	1	20	2	2	2	120	4	1
776	2	20	2	2	2	60	3	1
777	2	21	11	1	2	20	2	2
778	2	21	1	2	2	120	2	1
779	2	21	1	2	2	90	2	0
780	2	21	1	2	2	60	2	0
781	2	20	3	1	2	180	2	3
785	2	20	1	2	2	60	2	0
786	2	23	1	2	2	30	2	2
787	2	25	1	2	2	120	2	1
788	2	22	3	2	2	60	4	1
793	2	20	11	2	2	60	3	0
794	2	20	3	2	2	60	2	1
795	2	20	3	2	2	120	2	1
796	1	20	12	2	2	120	3	0
797	1	20	1	2	2	180	3	3
798	1	20	3	2	2	180	3	3
799	1	20	2	2	2	60	3	3
800	1	20	11	2	2	60	3	0
801	2	20	11	2	2	40	4	0
802	1	22	2	2	2	240	4	3
803	2	20	3	2	2	60	2	1
807	2	20	3	2	2	90	3	1

808	2	20	1	2	2	90	4	2
812	2	20	1	2	2	260	4	0
829	1	21	1	2	2	120	4	2
830	1	21	1	2	2	90	3	1
833	1	19	1	2	2	120	3	1
834	1	19	1	2	2	120	4	1
837	2	19	1	999	999	120	3	1
838	2	19	1	999	999	120	3	4
839	2	19	1	999	999	90	4	0
840	2	19	1	999	999	180	2	3
841	1	21	2	2	2	120	2	2
843	2	22	1	2	2	50	4	1
844	2	29	1	2	2	120	3	999
846	2	20	1	1	2	60	4	0
847	2	20	1	2	2	60	3	0
848	2	20	1	2	2	15	3	1
849	2	20	1	2	2	100	2	0
850	2	20	1	2	2	120	4	2
851	2	20	1	2	2	100	3	2
852	2	20	1	2	2	100	4	0
853	2	20	1	2	2	480	4	0
854	2	20	1	2	2	70	3	1
855	2	19	1	2	2	30	3	1
856	2	19	1	2	2	30	3	2
857	2	19	1	2	2	120	4	1
858	2	21	1	2	2	0	4	1
860	2	19	1	1	1	20	4	0
861	2	19	1	2	2	240	4	3
862	2	19	1	2	2	240	4	3
863	2	19	1	2	2	120	4	0
864	2	19	1	2	2	0	3	0
866	2	20	1	2	2	60	4	1
868	2	19	1	2	2	600	4	0
872	1	23	1	2	2	120	3	0
873	1	24	1	2	2	120	4	0
875	2	21	1	2	2	0	4	1
876	2	19	1	2	2	0	2	0
877	2	24	1	2	2	0	2	1
878	1	19	1	2	2	120	2	3
879	1	19	1	1	2	60	3	2
880	2	19	1	2	2	50	2	3
881	2	19	1	2	2	540	4	1
882	1	20	1	2	2	240	3	1
883	1	20	1	2	2	840	4	4
884	2	20	1	2	2	90	4	1
885	2	20	1	2	2	10	3	0
886	2	20	1	2	2	30	3	0
888	1	19	1	2	2	180	3	2
889	1	19	1	2	2	0	2	2
890	1	19	1	2	2	180	4	0

891	1	19	1	2	2	120	3	3
892	1	19	1	2	2	120	3	3
893	1	19	1	2	2	630	4	0
894	1	19	1	2	2	240	4	0
895	1	19	1	2	2	300	2	1
896	1	19	1	2	2	160	3	1
897	2	19	1	2	2	0	3	3
898	2	19	1	2	2	90	3	3
899	1	19	1	2	2	180	4	1
900	1	19	1	2	2	450	3	0
901	1	19	1	2	2	840	4	0
902	2	19	7	2	2	300	4	1
903	2	19	1	2	2	100	4	1
904	2	19	1	2	2	150	2	0
905	2	19	1	2	2	100	4	0
906	2	19	1	1	2	150	4	0
907	2	19	1	2	999	120	3	1
908	2	21	1	2	2	140	4	1
909	2	19	1	2	2	150	4	0
910	2	19	1	2	2	150	4	0
911	2	19	1	2	2	180	4	1
912	2	19	1	2	2	60	3	0
913	2	19	1	2	2	120	4	0
914	2	19	1	2	2	60	4	0
915	2	19	1	2	2	450	4	0
916	2	19	1	2	2	80	3	1
917	2	19	1	2	2	300	4	4
918	2	19	1	2	2	45	3	0
919	1	19	1	2	2	180	3	1
920	1	19	1	2	2	300	2	0
921	1	19	1	2	2	500	3	0
922	1	30	1	2	2	240	4	1
923	1	19	1	2	2	300	4	1
925	2	19	1	2	2	100	4	0
926	2	19	1	2	2	60	3	0
927	2	19	1	2	2	120	1	1
928	2	19	8	2	2	180	4	0
929	2	20	1	2	2	105	3	1
930	2	21	1	2	2	150	2	1
931	2	20	1	1	2	150	2	2
932	1	21	1	2	2	210	3	1
933	2	20	1	2	2	140	4	1
934	2	20	1	2	2	210	3	0
935	2	20	1	2	2	180	4	0
936	2	20	1	2	2	180	3	1
937	1	20	1	2	2	90	2	1
938	2	20	1	2	2	100	3	0
939	2	20	1	2	2	120	4	1
940	2	19	1	2	2	30	3	1
941	2	19	1	2	2	120	3	0

942	2	19	1	2	2	90	3	1
943	2	19	1	2	2	60	4	1
944	2	19	1	2	2	60	3	1
945	2	19	1	2	2	75	4	1
946	2	19	1	2	2	105	4	1
947	2	18	14	2	2	90	2	0
948	2	19	1	2	2	30	4	1
950	2	19	1	2	2	60	2	1
951	2	20	1	2	2	60	4	0
952	2	21	3	1	2	60	4	0
953	2	19	1	2	2	30	3	0
954	2	19	1	2	2	30	3	1
955	2	19	1	2	2	30	2	2
956	2	19	1	2	2	30	4	1
957	2	19	1	2	2	25	2	1
958	2	19	1	2	2	0	4	0
959	2	19	1	1	2	105	1	1
960	2	19	1	2	2	0	2	1
961	2	19	1	2	2	120	2	2
962	2	19	1	2	2	360	3	1
963	2	19	1	2	2	45	4	0
964	2	19	1	2	2	0	3	3
965	2	19	1	2	2	30	3	1
966	2	19	1	2	2	30	3	3
967	2	20	1	2	2	30	4	0
968	2	22	1	2	2	30	3	0
969	2	21	1	2	2	30	2	1
970	2	19	1	2	2	60	4	1
971	2	20	1	2	999	40	2	2
973	2	20	1	999	999	30	3	1
974	2	20	1	2	2	30	4	1
975	2	20	1	2	2	40	3	0
976	2	20	1	2	2	30	3	0
977	2	19	1	2	2	30	3	0
978	2	19	2	2	2	70	2	3
979	2	19	1	2	2	120	4	1
980	2	19	1	2	2	100	4	1
981	2	19	1	2	2	140	3	1
982	2	19	1	2	2	100	3	0
983	2	20	1	2	2	140	4	0
984	2	20	1	2	2	140	4	0
985	2	19	1	2	2	140	2	1
986	2	19	1	2	2	140	4	0
987	2	20	2	2	2	140	2	1
988	2	19	2	2	2	210	2	0
989	2	20	2	2	2	60	2	2
990	2	20	2	2	2	90	3	2
991	2	20	2	2	2	180	3	2
992	2	20	2	2	2	150	2	1
993	2	19	2	2	2	40	2	0

994	2	19	11	2	2	210	2	3
995	2	19	11	2	2	180	3	0
996	2	20	2	2	2	60	2	0
997	2	20	2	2	2	90	3	0
998	2	20	1	2	2	600	4	1
999	2	20	1	2	2	90	3	1
1000	2	21	1	2	2	210	4	1
1001	2	21	1	2	2	210	2	0
1002	2	20	1	2	2	210	3	0
1003	2	20	1	2	2	150	4	2
1004	2	20	1	2	2	60	2	1
1005	2	20	1	2	2	40	3	1
1006	2	20	1	2	2	40	4	0
1007	2	21	1	2	2	60	3	1
1008	1	20	1	2	2	450	4	0
1009	1	20	1	2	2	90	4	1
1010	1	20	1	2	2	360	4	0
1011	1	18	1	2	2	180	4	0
1012	1	18	1	2	2	180	4	1
1013	1	18	1	1	2	90	2	0
1014	1	18	1	1	1	90	4	0
1015	2	18	2	2	2	270	4	0
1016	2	20	1	2	2	60	3	4
1017	2	23	1	2	2	30	4	1
1018	2	20	1	2	2	360	3	0

C3	C4	C5	C6	C7	C8	C9	C10	C11	C12
4	3	3	2	4	0	4	1	3	1
4	4	3	1	4	1	4	2	3	3
3	3	3	3	3	3	3	3	3	3
3	4	4	3	4	1	4	2	3	2
4	3	3	3	4	0	3	0	3	3
2	2	2	1	3	1	2	2	2	2
4	4	4	1	4	1	4	2	3	1
4	3	3	1	4	0	4	2	3	1
1	4	3	3	4	1	2	2	4	2
2	1	2	1	2	0	1	1	1	1
3	2	3	1	4	2	4	3	3	3
1	1	1	1	2	0	3	1	1	0
4	1	3	1	4	4	4	1	3	1
3	2	2	0	2	0	2	0	2	3
4	4	4	0	4	0	4	2	4	0
2	1	2	1	3	2	3	1	1	1
2	1	2	0	3	0	2	0	2	1
4	2	3	3	3	2	3	2	2	2
3	2	3	4	4	1	3	2	3	4
1	2	3	1	3	0	2	1	1	2
4	1	3	0	4	0	4	3	4	0
2	1	2	2	4	1	3	3	4	1
3	2	3	1	3	1	3	1	3	2
4	2	4	4	3	3	4	4	2	3
3	2	2	1	3	1	3	1	1	1
3	3	1	1	2	4	3	3	1	2
3	2	3	4	4	1	3	1	2	3
2	2	2	1	3	1	2	1	1	2
3	2	2	2	4	1	2	1	3	2
4	2	4	2	4	0	4	1	4	1
3	2	2	1	3	0	2	2	1	0
4	3	3	3	4	0	3	3	3	3
2	2	3	2	3	1	2	1	1	2
4	2	3	0	4	0	4	0	3	0
4	1	2	0	2	0	4	1	1	0
4	3	4	1	4	0	4	2	3	1
3	2	1	2	3	4	2	2	999	3
4	2	3	1	3	1	3	1	2	2
3	2	2	1	3	0	3	2	2	2
2	3	1	1	4	2	2	1	1	1
4	4	4	3	4	0	3	1	2	0
4	4	4	4	4	0	4	2	4	4
1	2	3	2	1	2	2	2	3	2
3	3	2	3	3	3	3	2	2	2
4	2	3	1	4	1	4	2	3	1
4	2	3	2	4	0	3	1	4	4
4	3	2	3	4	0	3	2	2	3
3	3	2	1	2	1	3	1	2	1
3	1	2	2	3	1	3	0	1	2

2	1	2	2	3	2	3	1	2	1
3	3	3	2	2	2	2	1	3	3
3	2	2	3	3	2	2	3	2	3
2	0	1	3	3	0	2	0	0	4
3	1	2	3	3	2	3	2	3	3
3	3	2	0	3	0	3	0	1	0
2	1	2	3	3	0	3	4	2	3
3	2	2	0	4	0	4	1	1	0
3	0	1	0	1	0	2	0	1	1
3	3	2	2	3	2	3	2	3	3
3	3	2	0	2	0	2	0	2	3
4	3	3	2	4	1	3	3	3	2
2	2	2	2	4	0	2	2	2	2
3	3	3	2	3	0	3	2	3	1
2	2	1	3	3	1	2	1	1	2
4	4	3	1	2	0	4	4	4	1
2	3	3	3	3	3	2	2	2	3
2	1	1	1	3	0	2	1	2	0
2	2	2	1	3	0	3	1	2	2
4	2	2	3	4	1	3	1	2	2
4	4	4	1	4	1	4	2	2	3
3	2	2	1	4	1	3	1	2	3
4	4	2	1	4	1	3	0	1	0
2	3	2	1	3	2	2	2	1	1
2	2	2	2	3	1	2	1	2	2
2	3	4	1	3	0	3	0	1	0
3	4	4	2	4	0	4	1	3	1
4	2	4	2	4	0	4	3	4	2
1	1	0	2	3	0	2	0	0	1
3	4	4	4	3	3	3	2	2	2
4	4	4	0	4	0	4	0	4	3
3	3	3	2	3	0	3	2	2	2
4	1	3	3	3	1	4	1	2	2
3	3	4	2	4	2	3	1	1	1
4	3	4	2	4	1	4	2	4	3
3	2	2	0	4	0	4	1	0	0
3	0	3	0	4	0	3	0	0	0
4	2	2	1	3	0	4	1	2	1
4	2	1	1	3	1	3	0	2	0
3	2	2	1	1	3	2	0	2	2
4	4	3	0	4	3	4	4	4	0
3	2	2	0	4	0	4	1	2	1
3	3	2	2	4	0	2	2	2	3
3	2	3	2	4	0	4	1	2	3
3	1	2	3	4	0	2	2	2	3
2	2	2	4	2	3	3	1	1	4
999	3	4	3	4	0	3	1	3	2
4	2	2	1	4	0	2	0	3	1
4	3	3	2	3	1	4	2	3	3
3	2	3	1	4	2	4	3	3	3

3	3	2	2	2	3	3	2	3	2
1	1	1	1	4	2	1	4	1	1
4	2	2	1	4	1	4	2	3	1
4	4	4	0	4	0	4	4	4	0
3	2	1	3	3	2	3	1	1	2
1	4	2	1	0	1	4	1	2	2
2	2	2	2	3	0	2	1	1	1
4	2	3	0	4	0	4	2	3	0
2	1	2	1	3	0	2	0	0	0
2	2	3	1	2	1	2	1	2	1
4	3	2	2	4	1	4	2	2	3
4	2	3	0	4	0	4	1	4	0
3	2	3	1	3	1	3	2	3	1
3	2	3	2	3	4	3	4	3	4
3	2	3	4	3	3	4	3	3	2
3	3	3	2	3	1	2	2	3	2
0	4	4	0	0	0	0	0	3	0
3	3	3	3	3	2	3	2	2	3
3	2	3	1	3	0	3	0	3	3
3	2	3	1	3	0	3	1	2	2
2	2	3	3	2	3	3	2	2	2
3	0	2	1	3	1	4	0	2	1
3	1	3	2	4	3	3	2	3	3
4	2	1	3	1	2	3	4	1	2
4	1	3	0	3	0	3	0	1	0
3	2	2	1	3	1	2	2	2	3
3	1	1	1	4	3	2	3	2	2
4	2	3	0	4	0	4	1	4	2
3	3	3	3	3	3	2	2	2	2
3	4	4	1	4	0	2	3	3	0
3	3	3	2	3	2	3	2	3	2
3	3	4	4	4	3	4	3	4	3
3	2	3	3	4	3	2	3	3	3
3	3	4	3	4	3	4	3	2	3
1	1	1	1	1	1	2	1	1	1
3	1	2	2	3	1	3	1	1	1
1	1	2	0	2	1	3	2	2	1
4	4	3	4	4	4	3	4	4	4
4	4	4	1	4	1	4	3	4	3
4	3	4	4	4	3	4	3	3	3
2	2	4	0	4	0	4	0	0	0
3	3	3	3	3	3	3	1	1	2
3	3	3	3	3	3	3	1	1	2
3	3	3	3	3	3	3	1	1	2
3	3	3	3	3	3	3	1	1	2
3	3	3	3	3	3	3	3	1	1
3	2	3	3	4	2	4	2	3	2
3	2	3	2	3	1	3	0	2	2
3	1	1	1	3	2	1	0	1	1
3	2	0	1	4	0	4	3	0	0

2	2	1	1	2	2	3	0	0	0
4	1	0	0	4	1	2	3	2	0
1	1	1	1	4	1	2	0	2	0
2	1	3	1	2	4	2	1	3	2
2	1	0	2	3	2	1	2	1	1
2	0	3	1	4	4	2	0	1	1
4	4	3	3	4	3	3	3	3	4
2	2	2	1	3	0	2	1	1	1
3	3	3	3	3	1	3	1	3	3
3	3	3	3	4	4	4	4	4	4
3	3	3	0	3	0	3	0	2	2
2	2	2	2	2	2	2	2	2	3
3	4	4	1	4	0	3	2	3	1
2	2	3	3	3	4	3	4	3	4
3	3	3	0	4	0	4	1	3	0
3	3	2	3	3	2	3	2	3	2
4	1	2	4	4	0	4	0	0	3
3	1	3	2	3	2	3	2	2	2
2	2	2	3	3	1	3	3	2	3
2	1	1	3	4	0	2	1	1	3
3	2	2	1	4	0	3	2	3	0
3	0	3	1	4	0	3	2	2	1
4	3	3	2	4	2	4	0	3	4
3	2	2	2	3	1	2	0	2	1
3	1	3	2	2	3	3	2	2	2
2	0	0	4	4	0	4	0	4	3
4	4	3	2	3	0	3	1	3	0
4	3	4	1	2	1	4	1	4	3
4	2	3	1	4	1	3	1	2	2
4	3	3	2	4	3	4	2	3	2
4	2	1	1	3	2	2	1	1	1
2	1	1	1	2	1	2	2	1	1
1	1	1	1	1	1	2	1	2	1
4	2	4	1	4	1	4	1	4	1
3	2	2	1	3	1	2	2	2	2
3	2	3	3	3	3	3	2	3	3
4	1	2	3	4	0	3	2	3	3
4	1	2	3	3	0	2	2	3	3
4	1	2	2	3	0	2	2	2	3
4	1	1	1	2	4	1	1	1	1
4	1	1	1	2	4	1	1	1	1
1	2	1	1	3	2	1	1	1	1
4	1	1	1	3	3	4	1	1	2
4	3	3	3	4	0	3	2	4	2
2	4	3	1	3	1	3	3	3	2
3	1	2	2	2	1	2	1	3	2
3	2	1	0	2	2	2	0	1	0
3	2	1	1	4	1	3	1	1	3
2	2	2	3	3	1	2	2	2	2
2	0	2	2	2	0	1	0	0	0

4	4	1	3	2	4	4	4	4	4
3	1	1	1	2	2	2	2	3	2
4	4	4	4	4	1	4	4	4	4
3	3	2	2	3	2	3	1	1	2
3	3	3	2	3	0	3	3	3	3
3	2	3	3	3	1	3	2	3	3
3	1	1	0	3	0	2	0	1	1
4	2	4	0	4	0	4	0	3	0
2	0	1	0	2	0	2	0	1	0
3	0	1	3	3	1	2	0	1	3
4	0	0	0	1	0	3	0	0	1
4	4	4	0	4	0	4	3	3	1
2	2	3	3	3	3	2	3	2	3
4	3	3	3	3	3	4	3	4	3
4	2	3	2	3	3	3	2	3	3
3	3	2	2	3	3	2	2	3	2
3	2	0	0	0	1	2	2	2	2
0	0	2	0	3	0	2	0	1	0
1	1	1	2	1	2	1	2	2	2
3	1	3	3	2	1	3	1	4	3
3	2	3	3	3	2	3	1	1	2
4	3	4	0	4	4	4	0	4	0
3	1	2	2	2	1	1	1	1	1
3	3	2	2	3	1	3	1	3	3
4	4	4	0	4	0	4	4	4	1
4	2	1	1	4	1	4	2	2	0
4	1	2	1	3	0	4	2	3	1
2	3	2	3	4	1	3	3	3	2
3	2	1	1	999	3	3	1	1	1
1	1	0	0	4	0	2	0	0	0
3	3	3	2	4	0	3	2	3	2
2	2	3	0	3	0	2	0	1	1
4	1	1	1	3	0	4	2	2	1
1	0	1	0	3	1	2	0	1	0
4	2	2	0	2	0	4	0	0	0
3	4	3	1	3	1	4	1	4	1
4	1	1	0	4	0	4	0	0	0
3	3	4	0	3	0	3	2	3	1
4	1	2	1	3	0	4	2	3	1
2	2	2	2	2	2	3	2	2	2
3	1	2	1	3	1	3	1	1	2
2	1	2	1	3	1	3	1	1	2
2	2	3	2	3	1	3	0	1	0
3	1	3	1	4	1	4	0	2	1
3	3	3	1	3	0	2	1	3	1
4	4	0	4	4	0	4	0	3	0
4	2	2	1	3	0	4	0	2	0
4	1	3	2	3	0	3	1	2	2
3	1	3	1	2	2	3	2	2	1
4	3	4	3	3	3	3	3	4	4

4	4	4	4	4	4	4	4	4	4
4	3	2	3	3	2	2	3	3	2
3	0	4	0	4	0	4	0	4	0
1	0	1	1	2	1	1	0	1	2
3	1	4	0	4	0	3	4	3	1
1	0	0	0	0	4	0	0	0	0
4	2	2	3	4	1	3	2	4	3
2	1	1	1	3	1	3	0	1	1
2	2	2	2	2	2	2	2	2	2
4	0	2	3	2	3	3	3	2	4
4	4	3	3	3	4	4	4	3	3
4	2	3	0	4	1	4	1	1	0
4	4	3	3	3	4	4	4	3	3
4	3	4	1	4	1	4	1	2	1
3	2	4	1	4	1	3	1	1	1
3	2	2	3	3	2	3	1	2	3
3	3	2	2	2	2	2	2	2	3
3	1	1	2	3	1	1	1	1	1
3	2	4	2	4	1	4	2	3	1
3	1	2	1	3	1	3	1	2	1
3	2	3	1	3	1	3	2	3	1
4	1	4	1	4	1	4	1	4	1
3	1	3	1	3	1	3	1	3	1
3	1	3	1	3	1	3	1	3	1
4	1	4	4	4	1	4	1	4	4
2	3	2	3	3	3	2	3	2	3
4	0	3	3	3	1	4	4	4	4
4	3	2	1	2	0	4	0	0	2
1	1	1	1	2	2	2	0	0	0
2	1	2	1	2	2	1	3	1	2
3	0	3	0	2	0	2	0	3	2
2	1	2	2	3	2	2	2	2	2
3	1	3	2	4	1	4	1	2	1
2	2	3	1	2	3	2	4	2	2
2	3	2	1	2	1	3	2	3	2
4	3	4	3	4	4	4	3	3	3
2	1	2	1	3	2	3	0	2	0
3	3	3	0	3	1	3	2	3	0
2	3	2	0	2	0	3	1	2	0
2	3	3	1	4	3	4	2	2	2
4	4	4	4	3	3	4	3	4	4
3	4	3	1	3	2	3	3	3	4
4	4	4	4	4	4	1	4	4	4
2	1	2	2	2	2	2	3	3	1
3	2	2	0	3	0	2	1	1	2
3	4	4	4	4	0	4	4	3	4
3	2	2	1	3	0	3	2	2	1
3	4	4	4	4	0	4	4	3	4
3	3	3	1	3	1	3	2	3	3
3	3	3	1	4	1	3	2	2	2

0	4	3	4	3	0	0	0	0	2
3	2	2	2	3	2	2	2	2	2
3	3	3	2	2	1	2	1	3	2
1	3	2	1	3	1	3	1	2	1
3	3	3	1	3	1	3	1	1	1
3	0	0	0	4	0	3	0	0	2
3	1	2	1	1	1	2	1	1	3
4	4	4	0	4	0	4	4	4	0
3	3	3	1	3	1	3	3	3	3
4	3	3	1	3	1	4	3	3	4
4	2	3	2	4	0	4	1	1	2
3	1	2	0	4	0	4	1	3	0
4	4	4	0	4	0	4	1	1	4
2	4	2	3	1	3	1	3	3	0
3	3	4	3	3	4	3	4	3	3
2	1	1	1	2	1	3	1	1	1
3	2	2	1	3	0	3	2	3	3
4	2	2	3	3	0	4	0	3	3
3	1	1	1	4	0	4	0	1	1
2	2	2	2	3	1	2	2	2	1
3	3	3	0	3	0	4	0	0	0
4	2	3	1	4	0	4	2	2	0
3	1	2	2	3	2	2	2	3	3
3	2	2	4	4	2	3	2	2	4
4	1	2	1	3	0	3	1	4	4
2	2	2	2	2	2	2	2	2	2
4	1	3	2	3	1	3	1	2	2
2	2	3	0	3	0	3	0	2	0
2	2	2	2	3	0	2	2	3	2
3	0	3	0	4	0	4	0	2	0
3	0	1	0	4	0	1	0	1	0
3	2	2	2	3	1	4	2	2	2
4	4	4	4	4	1	4	1	2	1
3	4	3	0	4	0	4	2	3	1
3	4	4	4	4	1	3	4	3	4
4	3	3	1	3	1	3	2	3	2
4	1	2	3	3	1	4	1	2	2
1	2	3	1	3	4	3	1	2	2
3	4	4	2	4	1	3	2	1	4
3	2	2	2	3	1	3	1	2	1
3	1	1	1	3	0	2	1	2	2
4	3	2	1	4	1	2	1	1	1
4	0	3	0	3	0	3	0	2	3
1	0	0	2	4	2	2	0	0	2
3	3	3	3	3	3	3	3	3	3
3	0	2	0	4	2	2	0	0	2
4	4	4	0	4	0	4	4	4	0
3	2	2	2	2	2	2	2	3	3
1	0	2	2	3	2	2	2	3	2
4	3	4	3	4	0	3	1	1	3

3	4	2	2	2	4	4	4	3	3
3	2	3	2	3	3	2	4	3	4
4	3	3	0	3	0	3	2	4	1
4	3	4	2	4	0	4	0	2	2
3	3	3	1	4	1	3	1	3	1
2	3	2	2	2	1	2	1	2	2
2	0	1	1	2	1	2	0	1	1
2	2	2	2	1	2	2	2	2	2
3	2	3	1	3	1	3	2	1	2
3	2	2	1	3	1	3	1	1	1
3	1	2	0	4	0	4	0	2	2
3	1	2	0	1	1	3	0	0	0
3	1	2	0	1	1	3	0	0	0
3	1	2	0	1	1	3	0	0	0
2	0	2	0	4	0	2	0	2	1
3	2	3	1	3	1	3	2	1	3
3	2	3	1	3	1	3	2	3	1
3	3	2	1	2	1	3	1	2	1
2	1	1	1	4	0	3	0	2	0
4	3	3	1	3	1	3	2	2	1
3	2	3	3	3	1	3	3	3	4
3	2	3	0	4	4	4	2	2	1
3	1	1	1	3	0	1	0	0	0
0	2	1	4	3	0	1	0	0	2
4	2	2	2	4	0	4	0	0	0
3	3	3	2	4	1	4	2	3	3
4	4	4	2	4	0	4	2	2	2
2	0	1	1	2	2	2	1	1	1
4	4	3	3	4	3	4	3	4	4
2	0	0	0	4	2	2	0	0	0
2	0	0	0	4	2	2	0	0	0
2	0	0	0	4	2	2	0	0	0
2	0	1	1	2	2	2	1	1	1
4	4	3	3	4	3	4	3	4	4
4	1	3	4	4	1	4	1	3	3
2	1	1	1	2	2	2	1	1	1
2	1	1	2	3	0	1	0	2	0
2	1	1	2	2	2	2	0	1	1
4	2	3	1	4	1	4	1	4	1
4	4	3	3	4	3	4	3	4	4
2	999	1	1	2	1	1	1	2	2
2	1	3	2	4	0	2	1	2	4
2	1	2	1	2	0	1	0	0	1
4	4	4	1	4	2	4	2	4	2
3	2	1	1	4	1	4	2	2	1
2	0	2	3	3	0	2	2	3	3
2	1	4	1	2	1	3	1	3	3
1	1	2	1	4	2	1	1	1	3
3	2	2	3	3	0	4	0	2	2
3	3	2	2	1	1	3	2	3	1

3	2	2	2	1	3	2	4	1	2
4	3	3	1	4	1	4	2	3	2
3	3	3	0	3	0	3	2	2	0
4	4	3	3	4	3	4	3	4	4
2	3	2	4	3	3	4	3	2	2
2	2	3	2	3	2	2	2	3	3
2	2	3	1	2	1	3	1	3	1
2	2	3	3	2	2	2	1	2	3
1	1	2	0	1	0	0	1	1	2
2	2	2	2	2	2	2	2	1	2
2	0	1	2	3	2	3	2	2	1
0	1	0	0	1	2	1	1	2	1
1	1	1	1	1	2	1	1	1	1
2	2	1	2	2	2	1	2	2	2
2	2	2	2	2	2	3	2	2	2
1	1	1	2	2	1	1	2	3	2
2	1	2	2	3	1	3	1	1	2
2	2	1	1	2	0	2	1	1	2
2	2	2	0	4	0	2	0	0	0
3	4	3	1	4	0	4	0	2	1
3	1	1	1	2	1	2	1	1	1
3	2	3	3	4	1	3	2	3	2
4	4	4	3	4	0	4	3	4	0
3	2	3	1	3	1	4	1	3	1
4	4	4	2	3	0	4	1	3	3
4	3	3	0	3	0	3	1	1	1
2	1	2	1	3	1	2	2	2	2
4	2	3	1	4	1	3	1	2	3
4	1	4	1	4	1	4	0	1	1
3	2	2	3	2	1	3	1	2	3
4	2	2	1	4	1	3	1	2	2
3	2	2	3	3	3	3	2	3	3
2	2	4	4	4	3	3	2	3	4
4	4	4	3	4	0	4	4	3	4
4	3	3	1	4	1	4	1	4	3
4	3	2	2	2	2	3	1	1	1
4	3	3	1	4	1	4	1	4	3
4	4	4	3	4	0	4	4	3	4
2	2	2	1	3	1	3	1	1	1
2	3	3	1	4	2	3	0	1	1
3	1	4	0	3	2	3	1	3	2
2	2	1	1	3	1	2	1	2	1
4	4	4	4	4	3	4	2	4	3
4	3	3	0	4	0	4	3	2	4
2	2	2	0	2	0	3	3	2	1
1	2	2	1	3	3	4	0	1	0
1	0	1	4	0	1	0	0	0	3
4	1	3	0	3	3	4	0	1	0
3	2	1	2	3	2	1	2	4	3
3	4	3	2	3	2	1	2	3	4

3	1	3	0	4	2	3	0	3	1
3	2	3	2	3	2	3	4	4	2
4	0	4	0	4	0	4	0	4	0
4	3	3	3	2	3	4	3	2	4
3	1	2	2	2	0	2	1	2	2
2	0	0	1	4	0	2	0	0	1
2	1	3	2	3	2	1	3	4	2
3	1	2	2	4	2	3	1	999	2
3	3	3	1	3	1	3	1	2	1
3	1	2	0	3	1	4	3	1	2
3	2	3	2	3	2	3	2	3	2
4	4	4	2	4	1	3	2	2	2
2	3	3	2	3	4	4	1	1	4
3	0	3	1	4	1	2	0	0	0
4	4	4	0	4	0	4	1	4	0
4	2	2	3	4	1	2	0	1	2
4	3	3	1	4	0	4	2	4	1
4	3	2	1	3	1	3	2	2	2
2	1	2	3	3	2	2	1	1	1
3	2	2	2	3	0	3	1	2	2
3	3	3	1	3	0	3	3	2	1
3	4	3	2	4	0	3	1	2	1
3	1	2	2	4	1	3	1	2	1
0	2	2	0	2	0	2	0	2	2
4	0	0	2	3	2	2	0	0	2
2	1	1	1	2	1	2	1	1	1
3	2	3	1	2	1	2	2	3	1
4	3	3	2	3	2	3	3	3	3
3	2	3	2	3	2	3	2	3	4
2	1	1	1	2	1	3	2	3	3
2	1	2	1	3	1	3	1	2	2
2	1	1	2	2	0	2	1	1	2
2	1	3	2	2	0	2	1	3	2
2	3	2	2	3	2	2	2	2	1
2	1	3	2	2	0	2	1	3	2
3	2	2	3	3	2	3	2	2	3
2	1	2	1	3	1	3	1	2	2
2	3	2	3	2	1	2	1	2	1
3	4	3	1	3	1	3	4	3	2
2	1	1	3	2	2	3	2	2	2
2	1	1	3	2	2	3	2	2	2
3	1	2	2	3	3	3	1	3	3
4	0	2	1	1	1	2	1	2	2
4	0	2	1	1	1	2	1	2	2
4	4	3	4	4	4	4	4	4	3
3	1	2	2	3	3	3	1	3	3
4	2	3	1	4	0	4	2	4	2
2	3	4	3	3	3	4	3	3	4
2	1	1	1	2	1	2	1	1	2
4	2	3	3	3	1	2	2	2	2

3	1	3	1	4	2	4	1	4	1
4	2	3	1	4	0	4	0	4	0
4	1	4	1	3	2	4	1	4	3
3	2	4	4	4	1	4	2	3	1
3	1	3	1	3	1	3	1	3	1
4	1	4	1	4	1	4	1	4	1
3	1	3	1	3	1	3	1	3	1
4	1	4	4	4	1	4	1	4	4
4	0	4	0	0	0	4	0	4	0
2	3	2	3	3	3	2	3	3	3
2	2	3	3	3	3	3	3	3	2
4	4	3	3	2	4	2	3	3	4
0	4	4	4	4	1	2	3	3	3
3	1	2	4	4	0	3	1	3	0
3	2	3	0	2	0	2	1	3	1
2	4	3	4	3	2	3	4	3	4
4	0	2	0	3	0	4	3	2	4
3	2	3	1	3	0	4	0	2	0
3	1	1	0	3	0	3	1	2	0
4	2	3	3	3	0	4	2	2	2
2	1	3	2	3	0	1	0	3	1
4	0	1	2	3	1	3	2	1	1
2	1	2	2	3	1	2	2	2	3
3	3	3	2	3	2	3	2	3	3
3	2	1	1	2	3	2	1	1	1
3	3	4	2	3	0	3	2	4	4
4	4	3	4	4	4	4	3	3	3
4	4	3	3	4	3	4	3	4	4
4	4	3	3	4	3	4	3	4	4
4	2	3	1	4	1	4	1	4	1
2	1	1	2	3	0	1	0	2	0
4	1	3	2	3	1	3	1	2	2
4	4	3	4	3	4	4	3	3	4
4	3	3	0	4	0	3	0	1	1
1	3	3	0	4	0	2	2	2	0
2	3	2	3	4	4	4	3	3	4
2	1	1	0	2	999	2	1	2	0
2	2	2	2	2	2	2	2	2	2
4	2	2	2	3	3	3	4	2	4
3	2	3	3	2	4	3	2	3	3
4	3	2	3	4	3	2	4	4	4
4	2	2	4	4	1	3	2	1	3
4	4	4	1	3	1	4	1	2	1
4	4	4	4	4	4	4	4	4	4
4	3	2	3	4	0	4	1	3	3
4	3	2	2	3	0	3	2	2	2
3	2	2	2	3	0	3	1	2	3
3	4	2	3	4	2	3	4	2	3
4	3	2	2	2	3	4	4	4	3
4	4	4	1	4	4	4	1	3	2

3	3	3	2	2	3	4	4	3	3
3	3	3	2	2	3	4	4	3	3
4	4	4	0	4	0	4	4	4	0
4	4	3	2	4	0	3	3	3	1
4	3	4	1	3	2	4	2	3	2
2	3	2	3	3	3	1	2	3	3
3	3	3	2	2	3	4	4	3	3
3	3	3	2	2	3	4	4	3	3
4	2	4	4	4	2	4	1	4	4
4	1	3	2	4	0	4	1	4	0
4	1	3	0	4	0	3	0	3	0
4	4	4	3	4	1	4	2	4	3
3	2	3	1	3	1	3	2	2	2
4	3	2	2	4	0	4	3	2	3
3	1	2	2	4	0	4	1	1	2
4	2	4	1	2	0	3	1	3	0
4	4	3	3	4	1	4	2	3	3
3	2	3	2	3	2	3	1	1	1
3	2	2	2	3	1	4	3	2	2
4	2	3	2	4	0	3	1	1	0
4	4	4	1	4	1	4	3	4	4
3	2	3	1	3	0	3	2	2	1
4	4	4	0	4	0	4	0	2	0
3	3	3	3	4	1	4	0	2	2
3	3	2	2	3	0	3	2	2	2
2	0	1	1	2	2	2	1	1	1
4	0	2	2	4	3	4	3	1	1
4	2	2	0	4	0	3	2	2	2
2	2	3	2	4	1	2	2	2	1
3	0	3	0	3	0	3	2	3	0
4	4	4	1	4	4	3	4	3	4
3	4	3	3	4	2	3	3	1	3
1	3	3	0	3	1	2	2	4	3
2	4	4	2	3	1	4	2	2	1
4	1	1	1	3	0	3	0	1	1
4	4	4	3	3	1	4	2	3	3
3	3	3	3	4	0	4	0	4	3
3	2	2	2	3	2	3	3	1	2
4	3	2	3	2	1	2	3	1	4
3	1	1	3	4	0	2	0	2	2
3	1	2	2	3	1	3	1	2	2
4	2	3	3	4	1	4	2	2	1
3	3	2	3	3	0	3	3	2	3
4	4	3	0	4	0	4	3	3	0
1	2	2	2	3	1	3	1	1	2
3	1	3	2	2	1	3	1	1	1
3	3	3	4	4	0	4	4	3	3
4	3	3	1	4	1	4	1	4	3
4	2	3	2	3	1	4	2	3	3
3	4	2	0	4	2	3	1	2	1

3	1	1	1	3	1	3	1	1	1
2	2	3	3	3	1	2	3	3	3
4	3	3	3	3	1	3	2	2	2
3	2	2	1	4	1	3	2	2	2
2	2	2	3	3	2	3	1	2	3
4	4	3	0	4	0	4	2	2	0
3	3	4	2	4	1	3	2	3	2
4	2	3	1	4	1	4	1	1	1
4	3	3	3	4	0	4	2	2	0
1	1	3	1	2	0	2	0	0	0
4	2	2	0	2	0	3	2	2	0
2	2	2	2	3	1	3	1	1	1
1	3	1	3	0	3	0	3	2	1
4	3	4	2	4	1	4	4	4	3
3	1	3	3	3	1	2	2	2	3
3	3	2	2	3	0	2	1	2	2
2	1	1	3	1	4	1	1	3	3
3	2	1	0	1	2	2	1	1	2
4	2	2	1	3	1	4	1	1	1
2	2	2	1	3	1	3	1	1	1
4	3	3	0	4	0	4	2	3	0
3	1	1	3	3	3	3	1	1	3
3	2	2	2	2	1	2	2	2	1
3	2	2	3	3	2	3	2	2	2
2	4	3	0	4	0	4	2	3	2
2	2	3	0	3	0	3	0	2	0
2	1	2	1	3	1	2	2	1	1
4	3	4	1	4	1	4	2	3	1
4	3	3	3	3	3	3	2	3	4
2	1	1	1	3	1	3	1	2	1
2	3	2	2	2	1	3	3	2	3
3	2	2	0	3	0	2	0	1	0
2	2	2	1	4	0	3	2	2	1
3	2	2	1	3	0	3	2	2	1
3	0	0	1	3	3	3	0	0	1
4	1	4	3	4	1	4	2	4	2
4	2	3	0	4	0	3	1	3	3
1	1	1	2	2	1	3	3	2	1
4	2	3	2	3	0	4	0	3	3
2	1	2	1	3	0	4	1	2	3
2	3	3	4	4	0	4	4	3	4
3	1	2	4	3	1	3	2	1	3
4	4	4	3	4	0	4	2	4	3
2	2	4	3	3	1	2	1	2	2
1	2	1	1	3	1	2	1	1	2
3	3	2	3	3	2	3	1	2	4
3	2	2	2	3	1	3	2	3	3
3	1	2	2	4	2	3	1	3	2
2	1	3	2	3	1	2	0	1	2
1	2	1	1	3	1	2	1	1	2

2	2	999	3	2	3	3	0	0	2
2	2	2	3	3	2	3	3	2	3
2	1	1	1	1	1	1	0	0	1
2	1	3	0	3	0	3	0	4	0
3	4	3	2	3	1	3	2	2	1
2	2	2	2	3	1	3	1	2	2
4	3	4	4	4	4	1	2	2	4
3	1	2	1	3	0	4	0	3	0
3	1	3	2	3	3	3	1	1	3
3	3	3	1	3	2	3	2	2	1
2	2	2	3	3	1	3	4	3	3
3	3	2	2	3	1	3	2	2	3
2	4	0	3	4	0	2	0	0	3
3	3	2	2	3	1	3	2	2	2
3	4	3	2	3	3	3	4	4	2
4	2	4	1	4	1	4	2	4	1
4	3	4	0	4	0	4	3	3	4
1	0	2	0	2	0	2	0	0	0
3	2	3	1	3	2	3	1	2	1
3	1	1	0	2	0	2	1	2	4
1	0	2	0	2	0	2	0	0	0
2	2	4	1	3	0	2	1	3	1
4	3	4	3	3	2	3	2	2	2
3	4	3	3	3	1	2	0	0	0
3	2	2	2	3	0	3	2	1	1

C13	C14	C15	C16	C17	C18	C19	C20	C21	C22
4	0	4	1	4	1	2	0	3	0
4	0	4	3	3	1	3	0	4	2
999	1	4	4	3	1	2	1	3	2
4	0	3	1	2	1	3	0	4	4
4	0	4	3	3	0	1	0	4	2
2	2	2	2	2	2	0	2	3	1
4	1	4	2	3	1	4	0	4	3
4	0	4	2	3	1	1	0	4	0
4	0	2	2	3	1	2	1	3	2
2	0	2	1	2	1	1	0	1	2
3	0	3	3	3	2	3	0	3	2
2	0	1	0	1	0	1	2	3	1
4	1	4	4	4	3	1	1	3	3
3	0	2	2	2	0	2	0	2	2
4	0	4	4	4	0	4	4	4	4
2	1	2	1	2	1	1	1	2	1
3	0	3	1	2	0	1	0	3	1
3	1	3	3	3	1	2	1	3	2
4	1	2	2	3	2	3	0	2	2
2	1	2	2	1	0	1	0	3	2
4	0	4	2	3	0	0	0	4	3
2	3	3	1	0	3	3	4	2	3
4	1	2	1	2	1	1	1	3	2
4	1	4	3	3	4	3	1	3	2
1	0	3	4	2	0	2	1	3	3
1	2	3	1	2	0	1	1	3	4
4	0	3	3	3	1	2	0	3	3
3	1	3	0	1	0	1	1	2	1
2	999	3	1	2	3	2	1	2	2
4	0	4	4	4	2	4	0	4	4
3	0	2	1	1	0	2	1	2	1
3	0	4	3	3	2	2	0	3	3
2	0	3	1	3	3	2	1	3	3
3	0	4	0	3	0	0	0	4	2
4	0	4	2	3	0	2	0	4	3
4	0	4	4	3	0	2	0	4	2
2	2	2	1	2	3	3	3	999	2
3	0	4	1	1	2	3	0	4	1
3	0	3	1	2	2	2	1	3	1
4	1	2	1	1	1	1	1	3	1
3	0	4	1	2	0	1	0	3	0
4	0	4	2	4	0	2	0	4	2
1	2	3	2	3	3	2	3	2	2
2	2	2	1	1	0	1	3	3	2
4	0	4	3	3	1	2	0	4	3
3	0	4	1	2	0	0	0	3	1
3	0	3	3	2	1	0	0	3	2
4	1	3	2	4	0	0	0	3	1
2	0	3	0	1	0	0	0	1	2

3	2	3	1	1	1	1	1	3	1
4	4	4	3	2	2	3	3	2	2
2	2	3	2	2	3	2	3	2	2
3	0	2	0	0	0	2	0	2	0
2	3	4	4	4	3	3	3	3	2
4	0	4	4	2	0	4	0	4	4
2	0	3	2	2	0	1	0	3	1
4	0	3	3	3	0	3	0	4	3
1	0	3	1	1	0	0	0	3	0
3	0	3	1	3	0	2	0	3	1
2	0	2	3	1	0	3	0	2	2
4	1	3	3	3	2	4	1	3	3
3	0	3	1	2	0	1	0	2	2
2	1	3	2	2	1	1	3	2	2
2	1	3	1	1	2	2	1	2	2
4	0	4	4	4	1	4	0	4	4
2	2	2	3	2	3	2	3	2	3
4	0	2	2	1	0	1	0	3	1
3	0	3	2	2	1	1	0	3	1
4	1	4	2	2	1	2	2	4	2
4	2	4	3	4	1	2	1	4	2
3	1	3	2	2	1	2	1	3	2
4	0	4	3	2	0	2	0	3	2
4	0	3	0	0	0	0	0	2	3
4	1	3	1	2	1	2	1	2	2
3	0	3	2	2	0	1	0	3	2
4	0	4	1	3	3	1	0	4	1
4	0	4	3	4	3	3	0	4	3
3	0	2	0	1	0	2	1	3	1
3	0	4	3	3	2	3	0	2	2
4	0	4	1	4	0	4	0	4	0
3	0	4	2	2	1	2	0	3	3
3	1	3	1	3	1	1	1	4	2
3	1	4	3	4	1	3	1	4	2
4	1	4	4	4	1	3	0	4	4
4	0	4	1	1	0	1	0	3	1
4	0	3	999	2	0	0	0	4	0
2	1	3	3	2	1	2	1	3	1
3	1	3	3	2	2	2	0	3	3
2	1	2	1	1	1	2	1	3	1
4	0	4	4	2	0	3	0	4	1
4	0	4	2	1	3	2	4	1	1
4	1	3	3	2	1	3	3	2	3
4	0	4	1	3	0	1	0	4	2
3	0	1	3	2	1	1	0	3	0
2	1	2	1	1	1	2	1	2	1
4	0	4	3	3	2	3	0	1	3
3	0	3	0	4	1	2	0	4	0
4	0	4	3	3	2	3	0	4	2
3	0	4	2	3	2	3	0	3	2

3	3	3	2	2	3	3	1	2	2
4	1	2	1	1	1	1	3	2	1
4	1	4	4	3	4	4	1	3	1
4	0	4	3	4	0	4	0	4	4
2	1	3	1	1	1	1	1	3	2
4	1	4	2	2	1	2	1	3	1
3	0	3	3	2	0	1	0	3	1
4	0	4	2	3	0	3	0	3	1
4	0	2	0	1	0	0	0	3	0
2	1	3	1	1	1	1	1	2	2
4	1	4	3	2	2	1	1	4	2
4	0	4	2	3	0	2	0	4	2
3	1	3	1	1	1	2	1	3	3
3	3	3	4	3	4	3	4	3	4
3	3	4	3	3	3	4	3	2	3
3	1	3	1	3	3	3	1	3	2
4	2	0	1	3	0	0	0	0	0
3	3	2	2	2	3	3	3	2	3
3	1	2	2	3	1	2	1	3	2
3	0	3	3	3	1	2	1	3	2
2	2	2	2	2	3	3	3	3	2
2	1	3	1	1	2	0	0	3	1
2	3	3	2	3	1	3	3	2	4
3	2	4	1	3	2	1	2	3	4
3	0	4	0	1	0	0	0	3	0
2	1	3	2	2	1	2	1	2	2
2	3	3	2	0	2	1	1	3	2
4	0	4	0	2	0	3	0	4	2
3	3	3	3	3	3	3	3	3	3
4	0	4	4	4	3	2	0	4	4
3	3	2	3	2	3	2	3	2	3
2	3	4	3	3	3	2	4	3	3
4	3	4	3	4	3	999	2	3	4
4	3	3	4	3	4	3	4	3	4
2	2	2	2	2	1	2	2	2	2
1	1	1	1	1	1	1	1	3	3
4	0	3	3	2	0	1	0	4	2
3	4	4	3	4	4	4	0	4	4
3	1	4	4	4	1	4	0	4	3
3	4	4	3	3	3	3	3	2	2
2	0	2	0	0	0	0	0	4	0
3	1	3	1	3	1	1	1	3	1
3	1	3	1	3	1	1	1	3	1
3	1	3	1	1	1	3	3	3	3
3	1	3	1	1	1	3	0	1	3
2	3	1	3	3	1	1	1	3	1
4	2	4	3	4	2	3	2	4	3
4	0	4	0	2	1	1	0	3	0
3	3	4	0	0	1	1	0	1	1
3	0	3	1	0	1	4	0	3	4

3	1	3	0	2	1	0	2	1	1
2	0	2	2	3	1	0	0	2	2
4	999	0	1	1	1	0	2	0	1
3	0	3	1	1	0	0	1	4	2
0	0	1	2	2	1	2	1	2	0
2	0	2	0	0	0	1	2	4	1
4	3	3	3	3	4	3	4	3	4
2	0	2	1	2	0	1	0	2	1
3	1	3	3	3	1	2	1	3	1
3	3	3	3	3	3	4	4	4	4
3	2	2	2	3	3	3	3	2	3
3	3	3	3	3	3	2	3	2	4
4	0	3	3	4	0	4	0	3	3
3	2	3	2	3	2	3	3	3	2
3	0	2	3	2	0	3	0	3	2
3	3	3	2	3	3	2	3	3	2
3	0	4	2	4	0	2	0	4	0
3	2	3	2	2	3	2	3	2	3
4	1	2	2	2	2	2	1	2	3
4	0	4	2	1	0	1	0	2	1
4	0	3	2	3	1	2	1	3	3
4	0	3	2	3	0	4	0	3	3
4	4	3	2	3	4	3	2	4	3
4	0	3	2	1	0	1	0	2	1
3	1	2	2	2	2	2	0	3	1
4	0	3	0	2	0	2	0	3	2
3	0	4	3	3	0	3	0	4	4
4	1	4	2	4	1	1	1	4	1
3	0	4	2	3	0	3	0	4	3
3	1	4	1	3	1	2	1	4	1
3	1	4	1	1	1	1	1	2	1
2	2	1	2	1	1	1	1	1	1
1	1	3	1	2	1	1	1	3	1
4	1	4	2	4	1	4	1	4	2
3	0	2	2	2	1	1	2	2	2
4	1	3	4	2	1	3	1	3	3
4	0	2	2	2	3	3	0	3	3
3	0	2	2	1	3	3	0	2	3
4	0	4	2	2	2	3	0	4	3
2	4	3	1	1	1	1	2	2	1
2	4	3	1	1	1	1	2	2	1
2	2	1	2	1	1	2	1	2	2
4	2	4	1	1	1	1	1	4	1
4	0	4	2	4	0	3	0	4	3
4	1	4	2	3	1	2	0	3	3
3	2	2	3	2	1	1	1	2	1
3	2	2	0	1	1	0	2	2	1
4	0	3	0	2	0	0	0	3	0
2	1	3	2	2	2	2	2	2	2
2	0	0	0	0	0	0	1	0	0

4	0	4	0	2	0	3	0	1	0
3	2	2	2	3	1	2	1	1	1
4	1	4	1	4	1	1	1	4	1
4	0	3	2	2	0	2	0	3	2
4	0	3	3	3	2	2	0	3	3
4	0	4	3	3	1	3	0	4	3
2	0	2	0	1	0	1	0	2	0
3	0	4	1	2	0	1	0	4	1
1	0	2	0	1	0	0	0	2	0
1	2	3	0	1	0	0	2	2	0
2	0	4	0	0	0	0	0	3	0
3	0	4	3	3	0	3	0	3	2
3	2	4	4	2	3	3	0	3	3
4	3	2	3	4	2	3	1	3	3
4	2	3	2	3	2	3	3	4	2
3	3	3	2	3	2	3	2	3	2
2	2	2	3	2	2	1	2	2	3
3	0	2	0	1	0	0	0	2	0
3	1	1	2	1	2	3	2	2	1
4	0	4	3	2	0	3	0	4	3
2	2	3	3	3	3	2	2	3	3
4	0	4	2	4	0	2	0	4	2
2	1	2	2	3	0	1	0	2	1
3	0	3	0	2	0	1	0	3	1
4	0	4	4	4	0	3	0	3	3
4	0	4	3	2	0	3	0	4	3
3	0	4	2	3	1	2	4	4	2
3	0	3	3	3	1	3	1	3	3
3	1	3	1	1	1	1	1	3	1
4	0	2	0	0	0	0	0	2	0
4	0	3	3	3	2	3	0	4	2
4	0	3	0	2	0	3	2	3	3
2	0	4	2	2	0	2	0	4	2
2	1	2	0	0	0	0	1	2	0
2	0	4	0	3	0	0	0	3	0
3	1	4	4	3	0	1	0	4	3
3	0	3	0	2	0	1	0	3	0
4	0	3	3	3	0	2	0	3	3
3	0	4	2	3	1	2	0	4	2
2	2	2	2	3	2	3	2	2	2
3	2	3	2	2	3	2	2	3	1
2	1	3	2	2	1	1	1	2	2
2	1	3	1	3	0	0	0	3	1
3	0	4	0	2	0	0	0	4	0
2	0	3	2	2	1	2	0	3	1
0	0	4	0	3	0	0	0	4	0
3	0	4	0	3	0	4	0	4	2
3	0	2	2	2	2	2	0	3	1
3	2	3	2	2	2	1	1	2	3
2	4	4	4	4	3	4	4	4	3

4	4	4	4	4	4	4	4	4	4
2	3	2	4	3	3	2	3	3	2
4	0	4	1	4	0	4	0	4	0
1	1	1	2	1	1	2	1	3	2
4	0	3	2	3	0	4	0	3	3
0	2	0	0	2	0	0	0	0	0
4	1	4	3	3	2	3	1	4	3
3	1	3	0	0	0	0	3	0	0
2	2	2	2	2	2	2	2	2	2
2	3	2	3	3	3	3	0	3	1
4	4	4	3	3	3	4	4	4	3
2	0	4	0	1	0	0	0	4	1
4	2	3	3	3	3	4	3	3	4
4	1	4	1	3	1	1	1	4	1
3	1	4	1	3	1	1	1	4	1
2	2	3	2	1	3	1	2	3	2
3	3	3	3	3	3	4	4	4	4
3	1	4	1	1	1	1	1	4	1
4	1	3	2	4	1	1	1	4	1
2	1	3	1	2	1	1	1	3	1
3	1	3	1	3	1	2	1	3	2
4	1	4	1	4	1	2	1	4	1
3	1	3	2	3	1	2	1	3	1
3	1	3	1	3	1	1	1	3	1
4	1	4	1	1	1	1	1	4	1
2	3	2	3	2	3	2	3	2	3
4	0	4	0	4	2	0	3	0	3
4	0	4	0	2	0	0	0	4	0
2	0	2	0	0	0	2	0	2	0
3	2	1	3	2	1	2	2	1	3
3	2	0	0	2	3	2	2	3	2
2	3	1	1	2	3	1	1	2	1
3	0	4	1	3	1	1	0	4	1
2	3	3	3	2	3	2	3	2	3
2	1	2	2	3	2	1	1	1	2
1	3	4	3	3	4	2	2	3	3
3	0	3	3	3	0	2	0	3	3
3	1	3	2	3	0	3	0	3	3
2	0	3	4	3	0	2	0	4	3
3	0	4	1	2	1	1	0	3	1
4	4	3	4	4	4	4	3	4	4
4	4	4	0	3	0	2	0	4	3
4	1	4	4	4	4	4	1	4	4
1	1	3	3	3	3	3	3	3	3
3	0	3	1	2	0	2	0	2	0
4	0	3	4	3	4	4	0	4	4
2	0	3	2	2	0	2	0	3	2
4	0	3	4	3	4	4	0	4	4
3	1	3	3	3	1	2	1	3	3
4	1	3	2	2	1	2	0	3	2

2	3	3	999	2	2	4	2	3	4
3	0	3	2	2	0	0	0	2	2
3	1	3	2	3	2	1	0	2	2
3	1	3	2	2	1	2	1	3	2
4	1	4	3	3	1	3	1	3	3
4	0	3	0	0	0	0	0	3	0
3	3	3	1	1	3	3	3	3	3
4	0	4	4	4	0	3	0	4	4
3	1	3	3	3	1	3	1	3	3
4	1	4	3	4	1	3	1	4	3
4	0	4	2	3	1	1	4	4	2
4	0	4	0	3	0	2	0	4	0
4	0	4	3	3	0	2	0	4	3
3	0	3	1	2	0	2	0	4	1
3	3	2	3	2	3	3	2	3	4
2	1	2	1	1	1	1	1	3	2
3	0	4	2	3	0	2	0	3	2
4	0	2	0	3	0	3	0	3	3
4	0	4	0	1	0	0	0	4	0
4	1	2	2	2	1	2	1	2	2
3	0	3	2	2	0	0	0	3	2
4	0	4	2	3	0	2	0	4	2
4	1	999	1	2	2	2	1	2	1
4	0	3	2	2	2	2	1	3	2
4	0	3	2	3	0	0	0	3	4
2	2	2	2	2	2	2	2	2	2
3	1	3	1	2	1	3	1	3	2
3	1	2	3	2	0	2	0	2	3
2	0	3	2	2	2	3	0	2	3
4	0	4	0	3	0	0	0	2	0
3	1	2	0	1	0	2	0	3	0
3	1	3	2	2	1	2	1	4	2
4	1	4	4	4	1	1	1	4	1
4	0	4	2	3	0	3	0	4	3
4	1	3	4	3	4	4	1	3	4
3	1	3	3	4	2	3	1	4	3
4	1	4	1	3	1	1	1	4	2
2	2	2	2	3	1	1	1	3	3
4	0	3	3	2	0	3	0	3	1
3	1	3	3	3	2	3	1	4	2
3	0	3	0	3	2	1	0	1	1
4	1	4	2	3	1	1	1	3	1
3	0	3	0	3	2	3	0	3	3
0	3	2	0	0	0	0	2	2	0
3	3	3	3	3	3	3	3	3	3
4	2	4	0	0	0	0	2	4	0
4	0	4	4	4	0	4	0	4	4
2	3	2	3	2	2	3	2	3	3
1	2	2	3	2	2	2	2	3	2
4	0	4	1	4	1	1	0	4	1

2	3	2	4	4	3	3	2	4	3
4	3	3	2	2	3	2	2	1	3
4	0	4	2	4	0	2	0	4	4
4	0	4	0	3	2	3	0	3	2
1	1	3	1	3	1	1	1	3	1
3	1	2	3	2	1	3	2	3	2
2	0	2	1	1	0	1	0	3	1
2	1	2	2	2	1	2	2	2	1
2	1	3	2	1	2	1	1	2	1
2	1	3	2	1	1	1	1	2	1
2	0	2	2	2	0	2	0	4	2
2	2	3	0	2	0	0	1	3	0
2	2	3	0	2	0	0	1	3	0
2	2	3	0	2	0	0	1	3	0
4	0	3	3	3	0	3	0	3	3
1	3	3	3	3	1	2	2	1	2
3	1	3	3	3	1	2	1	2	2
2	1	3	2	2	1	3	1	3	2
3	0	3	0	3	0	0	0	2	0
4	0	4	3	3	0	2	0	4	2
3	0	4	3	3	2	3	0	4	3
2	0	4	4	3	0	3	0	3	3
3	0	3	0	0	0	0	0	3	0
4	0	0	0	0	0	0	2	0	0
0	0	4	0	2	0	0	0	4	0
4	1	4	2	4	1	2	0	3	3
2	2	2	3	0	3	3	3	3	2
2	1	2	0	1	0	1	0	2	1
4	3	3	4	4	3	4	3	3	3
0	2	2	0	0	0	0	2	2	0
0	2	2	0	0	0	0	2	2	0
0	2	2	0	0	0	0	2	2	0
2	1	2	0	1	0	1	0	2	1
4	3	3	4	4	3	4	3	3	3
3	1	3	1	4	1	1	1	3	1
2	2	2	1	1	1	1	1	2	1
2	0	3	2	3	1	0	0	3	1
2	2	2	1	1	1	1	1	2	1
4	1	4	2	4	1	2	1	4	4
4	3	3	4	4	3	4	3	3	3
3	1	2	1	2	1	2	1	3	1
4	0	3	2	2	1	3	0	3	2
2	0	2	0	1	0	0	0	1	1
4	0	4	3	4	0	4	0	4	4
2	1	4	2	2	1	2	0	4	2
3	0	3	3	3	0	0	0	3	2
2	2	2	3	3	3	2	2	2	2
3	1	3	1	1	1	1	1	1	1
4	0	4	2	3	2	2	0	3	2
3	1	3	3	2	1	3	1	3	3

3	2	2	2	3	3	3	2	3	2
4	1	4	3	4	2	2	1	3	3
2	0	2	0	2	0	2	0	3	2
4	3	3	4	4	3	4	3	3	3
3	3	2	4	3	3	3	2	3	3
3	3	3	3	3	2	2	1	3	2
2	1	3	1	3	1	1	0	2	1
3	3	2	3	2	3	3	3	3	2
1	1	1	0	0	0	1	1	1	2
2	2	2	2	2	3	2	2	2	3
2	3	2	2	1	2	1	0	1	2
1	1	1	2	2	1	2	3	2	2
0	0	1	1	1	1	1	1	1	2
1	1	2	2	1	2	1	2	3	2
3	3	3	2	2	2	3	2	2	2
2	3	3	3	3	2	2	2	1	2
4	0	3	2	2	1	1	0	2	1
3	0	3	3	3	3	3	0	3	4
4	0	3	0	2	0	1	0	4	0
4	0	4	4	2	0	4	0	4	4
2	1	2	1	2	1	1	1	2	1
3	1	3	2	2	1	2	1	3	2
4	0	4	2	3	0	3	0	3	3
3	1	3	2	3	1	2	1	3	1
3	3	3	3	4	0	3	0	4	2
3	1	4	4	3	0	2	0	3	2
3	0	2	1	1	0	0	0	2	1
4	1	3	2	2	2	3	1	4	3
0	0	4	2	3	2	2	0	0	0
3	2	3	1	2	0	0	1	3	2
3	1	4	1	3	1	3	1	4	1
2	3	3	4	3	2	3	3	3	3
4	2	4	4	4	4	4	4	4	2
3	4	3	0	4	2	4	0	4	4
3	0	4	3	4	1	3	0	3	3
2	1	3	1	2	2	2	0	2	1
3	0	4	3	4	1	3	0	3	3
3	0	4	4	4	2	4	0	4	4
3	0	2	2	1	0	1	0	2	1
3	1	3	1	1	0	1	0	2	1
2	1	4	1	3	2	1	1	3	1
3	1	3	2	1	1	1	1	3	1
3	1	4	4	4	3	3	4	3	3
4	0	4	4	4	0	3	0	4	3
2	2	3	2	2	0	1	2	1	3
2	1	1	2	1	0	2	0	3	3
0	2	0	1	0	1	0	1	0	0
3	2	3	0	3	0	2	0	2	1
4	2	3	2	3	2	1	1	2	2
3	2	3	4	2	3	2	2	1	2

3	0	3	1	3	0	1	0	3	0
2	1	1	2	1	2	3	3	2	3
4	0	4	3	0	0	2	0	4	2
3	3	3	4	3	3	3	3	3	3
2	0	2	1	2	1	1	0	4	2
0	3	2	0	0	0	0	3	2	1
1	3	1	3	2	1	4	1	1	3
4	1	3	0	2	2	1	1	2	1
3	1	3	2	2	1	2	1	3	2
3	1	3	0	2	0	0	1	2	3
3	2	3	2	3	2	3	2	3	2
4	1	4	2	2	1	2	1	3	3
2	1	1	0	2	1	0	1	1	1
2	1	2	0	2	0	1	0	3	0
4	0	4	0	4	0	4	0	4	4
2	0	2	2	2	1	2	0	2	2
4	0	4	2	3	1	4	0	4	4
3	0	4	2	2	2	3	0	3	2
2	1	1	1	1	2	1	1	3	1
2	0	3	2	2	0	0	0	2	1
3	0	3	4	3	1	2	0	3	3
4	0	3	3	2	0	3	0	4	4
2	1	3	1	2	1	1	1	3	1
2	2	2	1	1	2	2	2	2	1
3	0	2	0	0	0	0	2	3	0
1	2	2	0	2	2	1	3	2	1
3	1	3	2	2	1	2	1	3	2
3	1	1	3	3	3	3	1	3	3
3	1	3	2	3	3	2	2	3	2
3	2	3	2	1	2	1	1	3	2
1	3	4	2	3	1	1	2	3	2
2	1	0	1	2	1	1	1	2	2
1	3	3	1	4	4	1	2	0	2
3	1	2	2	1	1	1	1	1	1
1	999	3	1	4	1	2	0	2	1
2	2	3	2	2	2	2	2	3	2
1	3	4	2	3	1	1	2	3	2
4	1	3	2	1	1	4	1	4	4
4	0	3	4	3	2	4	0	4	4
1	3	3	2	1	2	2	1	2	3
1	3	3	2	1	2	2	1	2	3
3	1	4	1	3	1	3	0	2	1
2	1	2	1	2	1	1	0	2	1
2	1	2	1	2	1	1	0	2	1
4	3	2	4	3	3	3	3	4	3
3	1	4	1	3	0	2	1	3	1
4	0	4	2	4	0	3	0	4	3
3	3	3	3	3	3	3	3	3	4
3	0	2	3	1	0	3	0	3	3
1	1	4	2	3	4	2	1	3	2

4	2	4	2	4	2	1	2	4	3
4	0	4	2	3	0	2	0	3	3
4	1	4	2	4	3	2	3	1	2
4	1	3	2	4	1	1	1	4	1
3	1	3	1	3	1	1	1	3	1
4	1	4	1	4	1	2	1	4	1
3	1	3	2	3	1	1	1	3	2
4	4	1	4	1	4	1	1	1	1
4	0	2	2	4	1	2	1	4	1
2	3	2	3	2	3	2	3	2	3
2	2	2	2	2	3	2	3	3	3
3	2	4	3	4	3	2	2	3	3
2	0	3	1	4	0	999	0	4	4
3	0	4	2	3	0	3	0	4	4
2	0	3	2	3	2	1	0	2	2
3	3	2	4	3	4	3	4	3	2
3	0	4	1	3	0	1	0	4	0
2	0	4	0	3	0	0	0	4	2
1	1	3	1	1	0	1	0	2	1
3	0	3	3	3	2	3	3	3	3
3	0	3	1	2	0	0	0	3	1
2	1	4	1	1	0	1	0	1	1
3	1	3	2	2	1	1	0	3	2
2	2	2	2	3	2	3	3	2	2
2	1	3	1	1	1	1	1	2	1
4	0	4	2	4	2	1	0	4	2
3	3	4	4	4	4	4	4	4	4
4	3	3	4	4	3	4	3	3	3
4	3	3	4	4	3	4	3	3	3
4	1	4	2	4	1	2	1	4	4
2	0	3	2	3	1	0	0	3	1
3	1	2	1	2	3	1	1	3	2
4	0	4	3	3	3	3	0	4	3
2	0	1	0	0	0	0	0	3	0
4	0	3	2	3	0	1	0	2	2
4	3	3	4	4	3	2	1	3	2
2	0	3	1	1	1	1	0	2	0
2	2	2	2	2	2	2	1	2	2
3	3	3	3	2	3	3	2	4	2
4	2	2	3	3	4	3	2	3	3
3	3	2	2	2	3	3	4	3	3
4	1	3	2	2	1	4	1	3	2
3	1	4	2	3	1	3	1	4	3
4	999	4	4	4	4	4	999	4	4
3	0	3	2	3	1	2	0	4	2
3	0	3	2	3	2	2	0	3	2
3	0	3	2	3	0	0	0	2	0
2	3	2	3	2	3	2	3	4	2
2	1	1	3	3	2	3	4	4	3
4	0	3	3	3	1	4	0	4	2

3	0	3	3	3	3	3	0	3	3
3	0	3	3	3	3	3	0	3	3
4	0	4	3	4	0	4	999	4	4
4	0	4	3	3	0	4	0	4	4
2	4	2	3	1	3	2	4	1	3
2	1	2	2	1	2	3	1	2	2
3	0	3	3	3	3	3	0	3	3
3	0	3	3	3	3	3	0	3	3
4	2	4	1	4	1	2	2	4	2
4	0	4	2	4	1	3	0	4	1
3	0	4	0	2	0	0	0	3	0
4	1	4	3	4	1	2	1	4	3
4	1	4	2	3	1	2	1	2	2
4	0	4	2	2	1	2	0	4	2
4	0	1	1	0	0	0	3	1	2
3	0	4	1	3	0	2	0	4	2
4	1	4	3	3	1	2	1	999	999
2	1	3	2	1	1	1	1	3	2
3	0	3	2	3	0	1	0	3	2
3	0	4	3	2	1	3	0	4	3
4	1	4	2	3	1	3	1	4	3
4	0	4	2	2	1	2	0	3	1
4	0	4	3	4	0	2	0	4	0
3	0	3	4	2	2	1	0	3	3
2	0	4	3	3	1	3	0	3	2
2	1	2	0	1	0	1	0	2	1
4	0	4	0	0	0	0	0	4	0
3	0	3	2	2	0	0	0	3	1
4	2	3	3	1	2	2	1	3	2
3	0	3	2	3	0	2	0	3	2
4	0	4	4	4	0	4	0	4	3
3	2	3	4	1	3	3	2	3	3
4	2	2	3	4	0	3	1	2	3
3	0	3	2	3	1	2	3	2	3
3	0	4	2	2	1	1	0	3	2
1	1	4	2	4	0	3	1	4	2
4	0	4	1	3	0	0	0	3	0
3	0	3	2	1	1	1	0	2	2
3	1	3	1	2	1	2	1	4	2
4	0	3	1	1	0	1	0	2	1
4	1	4	1	2	1	1	1	4	0
4	1	4	3	3	1	2	1	4	3
3	0	3	4	3	1	2	0	4	3
4	0	4	3	3	0	4	0	4	3
3	0	1	1	1	1	2	1	3	1
2	1	3	1	3	1	1	1	3	1
4	0	4	3	3	2	3	0	4	4
4	1	4	3	3	1	2	1	4	2
2	0	4	2	2	0	0	0	3	0
3	0	4	2	3	0	1	1	3	0

3	1	3	1	1	1	1	1	3	1
4	0	2	3	3	3	3	1	2	3
3	1	3	2	2	1	2	0	3	3
4	1	4	2	2	3	2	1	4	2
3	1	4	2	4	1	1	4	3	3
4	0	2	3	4	0	2	0	4	4
3	1	3	3	3	1	2	1	3	3
3	1	4	1	2	1	2	0	4	0
4	0	4	2	2	2	3	0	4	4
4	0	2	1	0	0	0	0	1	0
3	0	3	2	2	0	1	0	4	2
2	1	3	2	2	1	2	0	1	1
4	0	2	0	0	1	0	0	3	1
4	1	4	3	4	3	4	1	4	4
4	1	3	2	3	2	2	1	4	3
3	0	4	2	2	0	1	0	3	2
3	1	2	1	1	1	1	4	1	1
1	1	2	1	1	1	1	0	2	1
3	1	4	2	1	1	1	1	2	1
3	0	3	1	1	0	2	0	3	1
4	0	4	4	4	0	3	0	4	4
3	2	4	2	2	2	2	1	2	3
3	1	3	1	2	2	1	1	2	2
2	3	3	2	2	2	2	2	3	2
4	0	4	2	3	0	2	0	3	2
3	1	2	3	2	0	2	0	2	3
3	1	2	1	1	1	1	1	2	1
2	1	4	3	3	1	3	1	3	3
3	3	3	3	4	3	3	3	3	3
4	1	2	1	2	1	2	1	2	1
4	1	3	3	2	2	4	0	3	2
3	0	3	1	1	0	0	0	2	0
3	0	2	2	2	0	1	0	2	2
3	0	3	2	2	0	1	0	2	2
2	3	3	0	0	1	0	1	3	0
4	1	4	2	3	2	3	1	4	1
4	0	3	1	3	4	1	0	3	2
3	1	3	2	1	1	1	1	3	2
4	0	4	3	3	0	1	0	4	1
3	1	4	3	3	1	2	0	3	2
4	0	3	4	3	0	3	0	4	4
3	1	3	2	2	1	1	1	3	2
4	0	4	3	4	0	2	0	4	3
3	1	4	3	3	1	2	1	3	2
3	0	2	1	2	0	1	0	3	1
3	1	3	3	2	1	1	1	2	2
4	1	3	2	3	2	3	1	3	2
4	2	3	1	3	2	1	0	4	1
3	0	2	0	0	1	1	1	3	1
3	0	2	1	2	0	1	0	3	1

4	2	1	0	0	3	0	0	1	1
3	1	3	3	2	1	1	1	3	2
2	0	1	1	1	0	1	0	2	0
3	0	3	1	2	0	1	0	3	1
3	0	3	2	3	2	2	1	3	2
3	1	2	1	1	1	1	1	3	1
4	4	1	4	4	4	4	3	1	2
1	0	2	1	2	0	2	0	3	2
3	1	3	3	3	3	3	3	2	3
3	2	4	1	1	1	1	0	4	1
3	1	3	2	2	3	3	1	3	3
3	1	4	2	2	1	1	1	3	1
4	0	2	4	0	2	4	4	0	3
3	1	3	3	3	1	1	1	3	3
3	0	3	4	3	0	4	0	4	4
4	1	4	2	4	1	2	1	4	2
4	0	3	3	4	0	3	0	4	3
4	0	3	0	3	0	2	1	0	0
3	1	4	1	2	2	3	1	2	2
4	0	2	1	2	0	1	1	3	2
4	0	3	0	3	0	2	1	0	0
4	1	2	0	4	0	1	1	3	1
3	4	4	3	3	4	4	3	3	3
3	0	3	3	2	1	2	1	2	2
4	0	3	2	1	1	1	0	3	2

C23	C24
3	1
3	1
3	1
3	1
3	2
2	2
3	1
2	0
2	1
2	0
3	2
2	0
3	1
2	0
4	0
2	1
2	0
3	1
3	2
1	2
2	0
2	4
3	1
4	4
2	2
4	4
3	3
1	0
2	2
4	0
1	0
3	2
3	1
3	0
2	0
3	0
3	1
3	0
2	1
3	1
1	0
4	0
3	3
2	1
3	0
1	0
3	0
3	0
2	0

3	2
2	2
3	3
2	3
3	1
2	0
2	0
3	0
0	0
3	1
3	0
3	1
2	2
1	2
1	2
3	0
1	2
1	0
2	0
2	1
4	4
3	2
1	0
3	0
2	1
3	0
3	0
4	0
2	2
3	3
4	0
2	1
2	2
2	2
4	4
2	0
0	0
2	0
1	1
2	1
3	4
3	0
3	4
3	0
1	3
2	1
3	1
2	0
3	1
3	1

3	1
1	1
3	2
4	4
1	1
2	1
1	1
2	0
0	0
2	1
2	1
3	0
1	1
3	4
4	3
2	1
0	0
2	2
3	0
3	0
2	2
3	2
4	3
2	3
3	0
2	2
3	1
4	2
3	3
4	4
2	3
4	3
4	4
3	4
2	2
3	3
2	1
3	0
4	1
2	2
4	0
3	1
3	1
2	3
3	1
3	1
3	2
2	0
2	0
4	2

1	0
3	1
2	0
3	0
1	1
3	0
4	4
2	1
3	1
4	3
3	2
4	3
3	0
2	2
2	3
3	3
2	0
2	3
3	1
1	0
2	1
3	0
4	3
1	0
1	2
3	2
3	0
1	4
3	0
3	1
1	1
2	1
1	1
4	1
2	1
3	3
2	3
3	3
3	3
1	1
1	1
1	1
2	3
4	1
3	0
2	1
1	1
3	1
2	2
0	1

0	0
2	2
3	3
2	0
3	2
3	1
1	0
2	0
1	0
1	2
1	0
3	1
3	1
3	2
3	2
3	3
2	2
1	0
1	3
4	1
3	3
3	0
3	2
3	0
3	0
2	1
3	1
2	3
1	1
0	0
3	0
2	0
2	0
1	0
1	0
3	0
0	0
2	0
3	1
2	3
2	1
2	2
2	0
2	0
2	1
4	0
3	0
2	1
2	1
3	3

4	4
3	3
4	0
3	2
3	0
0	0
2	1
0	0
2	2
3	2
4	3
2	2
4	3
3	1
3	1
2	3
2	2
3	1
3	1
3	1
3	1
4	1
3	1
3	1
4	1
2	3
3	0
2	2
2	0
3	2
2	0
2	2
4	1
2	3
2	1
4	4
3	0
3	1
2	0
2	0
4	4
3	1
4	4
3	3
2	0
3	3
2	0
3	3
2	1
2	1

3	4
2	1
3	2
2	0
1	1
0	2
3	3
4	0
3	1
2	3
2	1
0	0
4	0
3	0
3	2
2	1
3	3
3	0
3	0
2	1
2	0
1	0
2	1
3	4
3	2
2	2
3	1
2	0
2	2
1	0
1	0
3	1
4	1
3	0
3	4
3	1
2	1
2	2
3	3
2	1
2	0
2	1
4	4
0	0
3	3
2	0
4	0
3	3
2	2
0	0

2	3
2	3
4	0
3	4
3	1
2	1
2	0
2	2
1	2
2	1
2	0
1	0
1	0
1	0
3	0
2	1
2	1
2	1
2	0
3	0
3	0
3	0
1	0
0	2
0	0
3	1
4	3
2	0
4	3
1	0
1	0
1	0
2	0
4	3
3	4
1	1
2	0
1	1
4	1
4	3
2	1
2	0
1	0
3	0
2	2
3	0
2	2
1	2
3	2
3	1

2	2
4	2
2	0
4	3
3	2
1	3
3	2
3	3
1	1
3	1
3	2
1	2
1	2
2	3
3	2
2	3
2	1
2	2
2	0
3	0
2	1
3	1
4	1
3	1
4	0
2	0
2	0
3	1
0	0
2	1
4	1
2	3
4	2
3	0
3	1
3	2
3	1
3	0
0	0
1	0
4	1
2	1
3	4
3	0
1	2
2	0
1	0
3	0
3	4
3	3

3	0
4	3
4	0
4	3
2	1
1	0
1	3
2	0
2	1
4	1
2	3
2	1
2	1
3	0
4	0
2	1
2	1
3	3
1	1
2	1
3	0
2	0
2	1
2	2
0	0
1	0
3	1
3	3
3	3
2	3
3	3
2	0
1	3
1	1
3	1
3	2
3	1
3	1
3	0
2	1
2	1
3	1
2	0
2	0
4	4
2	2
4	0
3	4
2	0
4	1

4	1
2	0
3	0
3	1
3	1
4	1
3	1
4	1
4	1
2	3
3	3
4	3
4	0
4	0
2	3
4	3
3	0
3	0
2	0
3	0
3	1
1	2
2	0
3	2
1	1
4	1
4	4
4	3
4	3
4	1
2	0
3	1
4	0
0	0
3	0
3	2
2	2
2	1
3	3
2	3
4	3
2	1
3	1
4	3
3	0
3	0
2	1
3	2
2	3
3	0

3	0
3	0
4	0
3	0
1	2
2	2
3	0
3	0
4	4
4	1
4	0
4	4
2	1
2	1
0	1
1	1
999	999
2	1
2	2
3	0
4	1
2	0
4	0
3	0
2	0
2	0
2	1
3	0
3	1
3	0
3	0
3	3
4	1
1	3
2	0
3	3
3	0
2	0
2	3
1	0
2	1
3	1
3	1
3	0
2	2
3	1
4	0
3	1
2	1
1	0

2	1
3	3
1	1
3	1
2	1
2	0
3	2
1	0
3	0
1	0
2	0
1	0
1	1
4	2
3	1
1	2
1	1
2	1
2	1
2	2
3	0
2	3
2	1
2	2
3	0
2	0
2	1
3	1
3	3
1	1
2	2
1	0
2	0
2	0
0	1
3	1
4	0
3	3
1	0
3	2
2	0
2	2
4	4
2	2
1	1
2	3
3	2
3	2
3	1
1	1

0	0
2	3
1	0
2	0
2	2
2	2
4	4
3	0
3	3
1	3
4	3
2	1
4	1
3	1
4	4
4	1
3	0
3	0
3	1
2	0
3	0
2	2
4	4
1	1
3	1
