# Supplementary material for: Psychometric properties of the Malay version of the Behavioural Regulation in Exercise Questionnaire (BREQ-3)
Source: PLoS One. 2022 Jun 24;17(6):e0269099. doi: 10.1371/journal.pone.0269099 (PMC9231722; doi:10.1371/journal.pone.0269099)
Supplement: Attachment — Submitted filename: Response to Reviewers.docx [file pone.0269099.s002.docx]

Dear Mohammad Asghari Jafarabadi,

On behalf of my co-authors, I would like to thank you for your consideration of our manuscript, titled “Psychometric Properties of the Malay Version of the Behavioral Regulation in Exercise Questionnaire (BREQ-3)”. We thank the Editor and the Reviewers for the careful consideration of our manuscript and for the largely favourable appraisal of this work. We also appreciate the Editor’s and Reviewers’ comments and think that the comments substantially helped us enhance the paper’s overall quality. As such, we highly appreciate the opportunity to revise and resubmit the paper. We hope that we have now adequately addressed the Editor’s and reviewer’ concerns and that you will now find our manuscript a useful addition to PLoS One. However, should there be any further issues requiring our attention, we would be grateful for a further opportunity to rectify these. Below, you will find our detailed responses to the comments.

**Editor’s comments and authors’ responses:**

**Journal staff:**

1. Please ensure that your manuscript meets PLOS ONE's style requirements, including those for file naming. The PLOS ONE style templates can be found at

https://journals.plos.org/plosone/s/file?id=wjVg/PLOSOne_formatting_sample_main_body.pdf and

<https://journals.plos.org/plosone/s/file?id=ba62/PLOSOne_formatting_sample_title_authors_affiliations.pdf>

Response: We have ensured that the manuscript meets PLOS ONE’s style requirement.

2. Please review your reference list to ensure that it is complete and correct. If you have cited papers that have been retracted, please include the rationale for doing so in the manuscript text, or remove these references and replace them with relevant current references. Any changes to the reference list should be mentioned in the rebuttal letter that accompanies your revised manuscript. If you need to cite a retracted article, indicate the article’s retracted status in the References list and also include a citation and full reference for the retraction notice.

Response: We have added one reference (reference 2) and updated the reference list in the revised manuscript.

Reviewer #1:

1-Abstract: RMSEA and SRMR must be defined.

Response:

Thanks to the reviewer for pointing out our mistake. The full terms for the abbreviations have been spelled out in the abstract.

2- Introduction: "Insufficient physical activity is viewed as one of the most important risk factors for mortality worldwide" needs a reference.

Response:

References were added for the statement.

3- Introduction: The third paragraph: "Study showed" which study?

Response:

We deemed the sentence as unnecessary and hence removed the sentence to improve the clarity.

4- Introduction is relatively long.

Response: We have trimmed the introduction from 642 words to 536 words.

5- Methods: "There are 131 males" It might be better to change all tenses to "past".

Response:

We thank the reviewer for the comment. We have corrected the tenses accordingly.

6- USM needs a definition.

Response:

The definition of USM is available in Method section, Participants subsection, line 106.

7- "RMSEA" and "V" should be defined in the first place. They must also be defined at the bottom of table 2.

Response:

We have added the full terms for the abbreviations in the Method section, line 151-152. We have also added the definition below Table 2. Thanks for pointing out the omission.

8- "AVE" and "CR" must be defined in the text and at the bottom of tables 3 and 4 (not in the title).

Response:

The full terms for the abbreviations were spelled out in the Method section, line 193. We have also added the definition below Tables 3 and 4. Thanks for pointing out the omission.

9- There is no explanation about various Bs (B2, B6, ...). Specially in table 1.

Response:

Thanks to the reviewer. We have added the item explanation in Table 1.

10- "The composite reliability ranged between 0.746 – 0.841 for the five-factor model." How about the other models? If all 5 factors would not available, what will be the next step?

Response:

In this study, we calculated the composite reliability for the tested models reported in the manuscript. The composite reliability for six-factor model was mentioned in line 220 in the following sentence.

“In the six-factor model 2, the CR ranges between 0.449 and 0.841”. The composite reliability is also available in Table 2.

CR for six-factor model 1 is not needed as the model fitness has not been satisfactorily achieved.

We are not exactly understand what reviewer meant by “If all 5 factors would not available, what will be the next step?”. But we will try to answer in here. If the 5 factors is not available or not fit to the data in this study, we will discuss and get further opinion from the experts and/or contacted the original author of the questionnaire. We will not proceed with further item delection or removing subscale.

Reviewer #2: - Statistical analyses were done rigorously and appropriately.

- Discussions were made clear and results were properly explained.

- Writing style could be revised for better readability and consistency. Past tense, present tense and future tense were inconsistently used throughout the paper and within each paragraph.

Response:

We thank the reviewer for the comment. We have revised the use of tenses accordingly.

- Authors reported having received 674 responses; yet, also reported to have excluded responses (without reporting the number of removal) and leading to the final 674 data analyzed. This brings to an incongruency with the collection and removal of data reported, or confusion to the readers.

Response:

The details were added in the line 105-106 in the Method section.
