# Supplementary material for: Psychometric properties of the Malay version of the Behavioural Regulation in Exercise Questionnaire (BREQ-3)
Source: PLoS One. 2022 Jun 24;17(6):e0269099. doi: 10.1371/journal.pone.0269099 (PMC9231722; doi:10.1371/journal.pone.0269099)
Supplement: Attachment — Submitted filename: Response to Reviewers 020322.docx [file pone.0269099.s003.docx]

Dear Mohammad Asghari Jafarabadi,

On behalf of my co-authors, I would like to thank you for your consideration of our manuscript, titled “Psychometric Properties of the Malay Version of the Behavioral Regulation in Exercise Questionnaire (BREQ-3)”. We thank the Editor and the Reviewers for the careful consideration of our manuscript and for the largely favourable appraisal of this work. We also appreciate the Editor’s and Reviewers’ comments and think that the comments substantially helped us enhance the paper’s overall quality. As such, we highly appreciate the opportunity to revise and resubmit the paper. We hope that we have now adequately addressed the Editor’s and reviewer’ concerns and that you will now find our manuscript a useful addition to PLoS One. However, should there be any further issues requiring our attention, we would be grateful for a further opportunity to rectify these. Below, you will find our detailed responses to the comments.

**Editor’s comments and authors’ responses:**

Reviewer #2: Line 109-110: Recommend to mention the measures used to determine "sound reading and speaking skills in Malay", be it self-reported or any other methods. If none were used, please omit this line.

Response:

In this study, we did not use the measure or method to determine the reading and speaking skills. However, Malay Language is compulsory subject for all students in Malaysia in school and to pass the subject to enroll in public university. Therefore, all respondents had reasonable level of reading and speaking skills in Malay. Nevertheless, to avoid confusion and improve the clarity of the manuscript, we changed the line to “able to understand Malay language”.

Line 141-142: Would prefer a more congruent reporting given that you've reported information about "non-smokers" under the participants section.

Response:

Thank you for the suggestion. We have added “smoking status” in the measures section.

Overall: The use of tenses had been corrected accordingly. However, there are still some minor grammatical errors throughout the paper. Would be good to have it revised.

Response:

We thank the reviewer for the comment. We have revised the use of tenses accordingly.
